# Quintuply orthogonal pyrrolysyl-tRNA synthetase/tRNA^Pyl^ pairs

**DOI:** 10.1038/s41557-023-01232-y

**Published:** 2023-06-15

**Authors:** Adam T. Beattie, Daniel L. Dunkelmann, Jason W. Chin

**Affiliations:** 1 Medical Research Council Laboratory of Molecular Biology, Francis Crick Avenue, Cambridge, England, UK

## Abstract

Mutually orthogonal aminoacyl-tRNA synthetase (aaRS)/tRNA pairs provide a foundation for encoding non-canonical amino acids (ncAAs) into proteins, and encoded non-canonical polymer and macrocycle synthesis. Here we discover quintuply orthogonal pyrrolysyl-tRNA synthetase (PylRS)/ tRNA^Pyl^ pairs. We discover empirical sequence identity thresholds for mutual orthogonality, and use these for agglomerative clustering of PylRS and tRNA^Pyl^ sequences; this defines numerous sequence clusters, spanning five classes of PylRS/ tRNA^Pyl^ pairs (existing classes: +N, A, B, and newly defined classes: C, S). Most PylRS clusters belong to classes that are unexplored for orthogonal pair generation. By testing pairs from distinct clusters and classes, and pyl tRNAs with unusual structures, we resolve 80% of the pairwise specificities required to make quintuply orthogonal PylRS/tRNA^Pyl^ pairs; we control the remaining specificities by engineering and directed evolution. Overall, we create 924 mutually orthogonal PylRS/tRNA^Pyl^ pairs, 1324 triply orthogonal pairs, 128 quadruply orthogonal pairs, and 8 quintuply orthogonal pairs. These advances may provide a key foundation for encoded polymer synthesis.

## Introduction

The genetic code of living cells has been reprogrammed to enable the site-specific incorporation of non-canonical amino acids (ncAAs) and hydroxy acids into proteins, and the encoded synthesis of non-canonical polymers and macrocyclic peptides and depsipeptides.^1–4^ These advances are underpinned by the discovery of aminoacyl-tRNA synthetases (aaRSs) and tRNAs that are orthogonal – in their aminoacylation specificity – with respect to the synthetases and tRNAs of the host organism, and mutually orthogonal with respect to each other. Several of these pairs,^5–16^ have been altered to recognise distinct amino acids (Supplementary Note 1). While initial work incorporated ncAAs in response to the amber codon, recent work has taken advantage of other codons including additional stop codons,^17^ quadruplet codons,^18–21^ codons containing non-canonical bases^22–24^ and sense codons in organisms with genomic code compression and tRNA deletion.^3,25,26^ Mutually orthogonal pairs provide a foundation for incorporating combinations of ncAAs and encoded cellular polymer synthesis and, despite recent progress, the discovery of such pairs remains an outstanding challenge.^1,2,5,6,17,18,27–30^

The pyrrolysyl-tRNA synthetase PylRS/tRNA^Pyl^ pairs are the most widely used systems for genetic code expansion.^2^ These pairs enable the site-specific incorporation of ncAAs in all domains of life;^31^ the anticodon of the pyl tRNAs tested can be mutated to decode diverse codons,^6,19,21,32,33^ as it is not a recognition element for PylRS enzymes;^34^ and the PylRS active site does not recognise canonical amino acids and can accept, or be evolved to accept, diverse ncAAs and hydroxy acids.^4,10,35–40^

Most genetic code expansion work with pyrrolysyl systems has focussed on the *Methanosarcina mazei* (*Mm*)PylRS/*Mm*tRNA^Pyl^_CUA_ pair and the closely related *Methanosarcina* *barkeri* (*Mb*)PylRS/*Mb*tRNA^Pyl^_CUA_ pair.^31^ The PylRS enzymes of these pairs are composed of two domains: an amino (N)-terminal domain and a carboxy (C)-terminal domain. The C-terminal domain binds the amino acid substrate and catalyses the aminoacylation of the cognate tRNA^Pyl^, and the N-terminal domain contacts the variable and T loops of the tRNA^Pyl^ to enhance binding affinity and specificity.^34,41^ Both domains are required to create a functional *Mm*PylRS/*Mm*tRNA^Pyl^ pair in *E. coli*, and it was widely thought that all PylRS systems required both domains for activity.^42,43^ We demonstrated that a newly defined group of PylRS enzymes^12^ – ΔN PylRS, lacking an N-terminal domain (in the same polypeptide or in trans) – are active and orthogonal.^7^ These pairs, and their engineered derivatives, were combined with pairs from the canonical +N group to enable the creation of mutually orthogonal pyl systems. We further showed that PylRS and tRNA^Pyl^ sequences in the ΔN group clustered into two classes, A and B, on the basis of their sequence identity, and we created triply orthogonal pairs composed of a pair derived from the +N group, a class A pair, and a class B pair.^6^ The discovery of new mutually orthogonal pyl systems has been combined with strategies for providing codons with which to encode non-canonical monomers, and this has enabled the incorporation of several distinct non-canonical amino acids into a protein and the encoded cellular synthesis of non-canonical polymers and macrocycles.^3,7,21,28,44–48^ Despite these advances there were no criteria with which to effectively search genomic data for mutually orthogonal pyl systems and we hypothesized that many orthogonal and mutually orthogonal systems remained to be discovered.

Here we leverage experimental PylRS/tRNA^Pyl^ cross reactivity data to empirically define sequence identity thresholds for mutually orthogonal PylRS enzymes and pyl tRNAs. We then perform agglomerative clustering on 351 PylRS sequences, to define clusters of sequences that pass the empirical thresholds; 84% of the resulting clusters belong to PylRS classes that have not been explored in the search for orthogonal pairs. We identify and cluster tRNA^Pyl^ sequences from the same organisms for members of 95% of the PylRS sequence clusters. Using both the empirical orthogonality thresholds and the presence of exotic structural features that may confer orthogonality, we select a set of pyl tRNAs which, along with PylRS enzymes from the same organism, form the starting point of an experimental search for mutually orthogonal pairs.

We identify two new classes of PylRS and tRNA^Pyl^ sequences, which we name class C and class S, and we show that the majority of our PylRS enzymes and pyl tRNAs are active and orthogonal in *E. coli*. We explore the specificity of class S and class C systems with respect to each other and with respect to previously characterized class N, A and B PylRS systems. Strikingly our sequence-based approach allows us to control 20 of the 25 aminoacyl-tRNA synthetase/tRNA pairwise specificities required to make a set of quintuply orthogonal PylRS/tRNA^Pyl^ pairs without additional engineering; we control the remaining five specificities by tRNA^Pyl^ engineering and directed evolution. Overall, we create 924 mutually orthogonal PylRS/tRNA^Pyl^ pairs, 1324 triply orthogonal pairs, 128 quadruply orthogonal pairs, and 8 quintuply orthogonal pairs.

## Results

### Cross-reactivity and sequence identity of pairs

We previously defined the cross-reactivity profiles of PylRS/tRNA^Pyl^ pairs belonging to three distinct classes (N, A, and B); we showed that certain non-cognate pairs, drawn from the ΔN PylRS classes A and B, exhibit a surprising degree of natural orthogonality with respect to one another. We postulated that this mutual orthogonality might be related to the sequence identity between the pairs, and we therefore decided to draw on ΔN PylRS/tRNA^Pyl^ activity data^6^ to quantify this relationship.

**Fig. 1:**
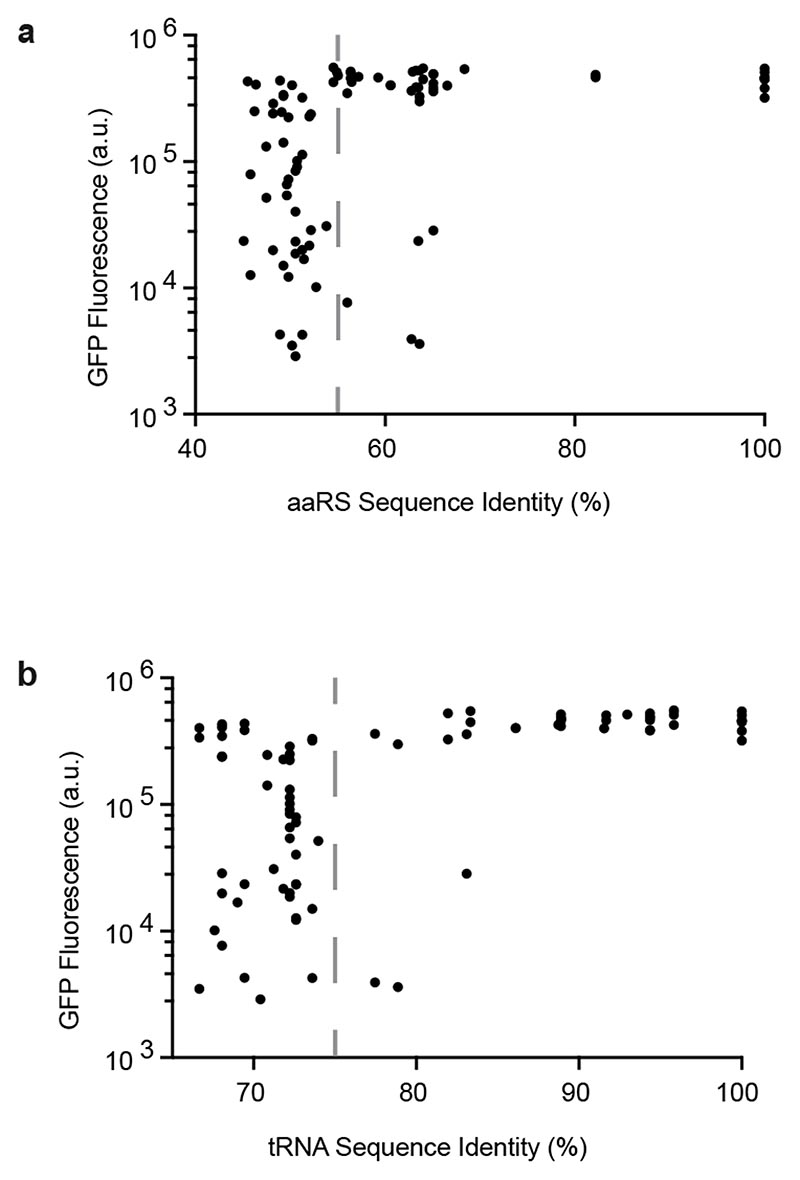
Relationship between sequence identity and cross-reactivity in previously characterized ΔN PylRSs and pyl tRNAs. **a**. Activity of each combination of ΔN PylRS_*i*_ and ΔN tRNA^Pyl^_*j*_, measured by production of GFP(150AllocK)_His6_ from cells bearing a *GFP(150TAG)_His6_* gene in the presence of 4 mM AllocK **1**, plotted against the sequence identity between ΔN PylRS_*i*_ and ΔN PylRS_*j*_, where ΔN PylRS_*j*_ is the synthetase from the same organism as ΔN tRNA^Pyl^_*j*_. ΔN PylRS proteins with greater than 55% sequence identity (dashed grey line) are predominantly active with each other’s pyl tRNAs (88% of cases). ΔN PylRS proteins with less than 55% sequence identity may or may not be active with each other’s pyl tRNAs. **b.** Activity of each combination ΔN PylRS with greater than*i* and ΔN tRNA^Pyl^_*j*_, plotted against the sequence identity between ΔN tRNA^Pyl^_*i*_ and ΔN tRNA^Pyl^_*j*_, where ΔN tRNA^Pyl^_*i*_ is the tRNA^Pyl^ from the same organism as ΔN PylRS_*i*_. ΔN pyl tRNAs with greater than 75% sequence identity (dashed grey line) are predominantly active with each other’s synthetases (93% of cases). ΔN pyl tRNAs with less than 75% sequence identity may or may not be active with each other’s synthetases. Dots represent the mean of three biological replicates, error bars are shown in Supplementary Figure 1. All numerical values are provided in Supplementary Table 2.

Strikingly, we found that if two ΔN PylRS enzymes had a sequence identity of over 55%, then one ΔN PylRS enzyme would show high activity with the tRNA^Pyl^ that naturally pairs with the other ΔN PylRS enzyme (or *vice-versa*) in approximately 90% of cases (Fig. 1a and Supplementary Fig. 1). Below 55% sequence identity, ΔN PylRS enzymes exhibited a range of activities with the pyl tRNAs of other ΔN PylRS enzymes. Similarly, if two tRNA^Pyl^ genes shared a sequence identity of over 75%, then one tRNA^Pyl^ would show high activity with the synthetase of the other tRNA^Pyl^ (or *vice-versa*) in approximately 90% of cases (Fig. 1b and Supplementary Fig. 1). Below 75% sequence identity, pyl tRNAs exhibited a range of activities with the ΔN PylRS enzymes of other pyl tRNAs.

This analysis suggested that the development of new multiply orthogonal pairs should focus on PylRS/tRNA^Pyl^ pairs whose synthetase and tRNA sequence identities are less than 55% and 75%, respectively.

### Identification and clustering of PylRS sequences

We assembled a database of PylRS sequences by performing a BLAST search for sequence similarity to the *Candidatus Methanomethylophilus alvus* (*Alv*) ΔN PylRS sequence (class A; henceforth referred to as A^Δ^-*Alv*PylRS). We retrieved 351 PylRS protein sequences (Supplementary Fig. 2 and Supplementary Table 1); of these, 79 belonged to the archaeal +N group, 66 belonged to the archaeal ΔN group, and 204 belonged to the bacterial (sN) group. In addition, two PylRS genes, despite being classified as archaeal, possessed a separately encoded N-terminal domain – we termed these the archaeal sN group.

**Fig. 2:**
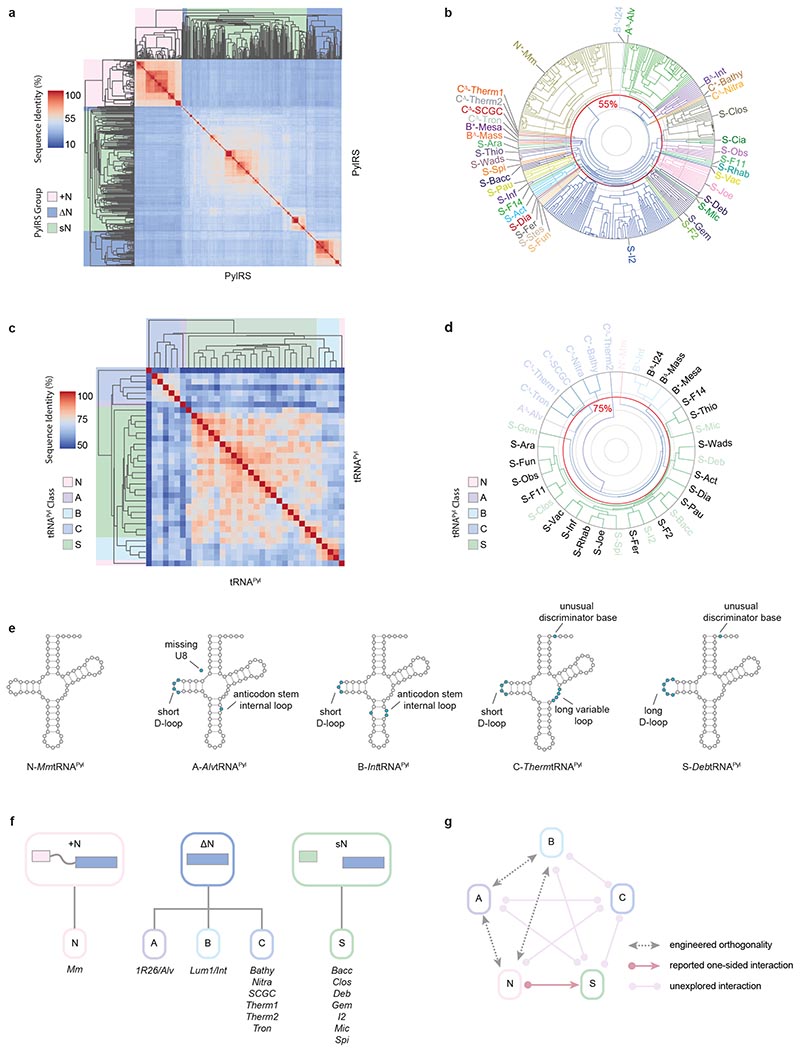
Selection of candidate PylRS and tRNA^Pyl^_CUA_ sequences and partitioning of the pyl system into five distinct sequence-defined classes. **a.** Clustergram of 351 PylRS C-terminal domain amino acid sequences retrieved. Three groups: +N (red), ΔN (blue), and sN (green) are shown over the dendrogram. Using a clustering threshold of 55%, 37 clusters were obtained. The heatmaps display percentage sequence identity scores. **b.** Dendrogram of the 37 clusters generated from agglomerative hierarchical clustering of the 351 PylRS C-terminal domain amino acid sequences. The 37 PylRS representative sequences are labelled. The radial coordinate represents percentage sequence identity (log scale). Grey contours 20% intervals, red contour 55% sequence identity, the clustering threshold value. **c.** Clustergram of 35 tRNA^Pyl^ sequences from the same organism as a representative PylRS from each cluster. The five pyl system classes are indicated over the dendrograms: N (red), A (purple), B (light blue), C (dark blue), and S (green). **d.** Dendrogram showing the eight clusters generated from agglomerative hierarchical clustering of the 35 identified tRNA^Pyl^ sequences. Coloured labels correspond to the 16 tRNA^Pyl^ sequences chosen for experimental characterization along with their cognate PylRS enzymes. The radial coordinate represents percentage sequence identity (log scale). Grey contours 20% intervals, red contour 75% sequence identity, the clustering threshold value. **e.** A representative tRNA^Pyl^ from each class is shown; notable structural differences with respect to the canonical N-*Mm* tRNA^Pyl^ in blue. **f.** Schematic of the three Pyl system groups and their division into five classes. Names of the Pyl systems chosen for characterisation are annotated below each class. For classes A and B, the A^Δ^-*1R26*PylRS/A-*Alvt*RNA^Pyl^ and B^Δ^-*Lum1*PylRS/B-InttRNA^Pyl^ pairs were used. For all other classes, PylRS/tRNA^Pyl^ pairs were derived from the same organism. **g**. Schematic of the interaction network between all five classes. Classes N and S are known to interact (red arrow); interactions between classes N, A and B can be abolished by tRNA engineering (grey arrows). All other interactions between classes are unexplored (pink arrows).

We performed an agglomerative hierarchical clustering to visualise the sequence diversity among PylRS catalytic domains (Fig. 2a and Supplementary Table 1). We observed two major groups: a dense cluster consisting of the class N PylRS sequences, and a loose cluster consisting of the bacterial and other archaeal PylRS sequences. The latter cluster itself contained several denser sub-clusters, including those corresponding to the known archaeal class A and B sequences. To discover mutually orthogonal systems, we focussed on identifying PylRS sequences with pairwise sequence identities of less than 55% (Fig. 1a). To achieve this, we set a linkage distance threshold for the agglomerative clustering such that two clusters would be merged if, and only if, the average of the percentage identities of each PylRS in the two clusters was greater than 55%. This led to 37 clusters (Fig. 2b, Supplementary Fig. 3, Supplementary Table 1). Three clusters represented the known PylRS classes N, A, and B. By contrast, there were 25 bacterial sN-group clusters and 9 further archaeal clusters (seven ΔN-group and two archaeal sN-group). This analysis demonstrated that substantial sequence diversity among Pyl systems remains to be explored.

### Identification and clustering of tRNA^Pyl^ sequences

For a representative PylRS enzyme from 35 of the 37 clusters we identified the corresponding pyl tRNA gene from the same organism^49^ (Supplementary Table 1).

We performed an agglomerative hierarchical clustering of the 35 tRNA^Pyl^ sequences (Fig. 2c). We observed tighter grouping than with the PylRS sequences. Of the nine identified archaeal pyl tRNAs, three grouped with class B and six formed a clear albeit more loosely related grouping, which we termed class C. Meanwhile, bacterial sN pyl tRNAs grouped together strongly; we assigned these to a new class S. Curiously, the pyl tRNAs of the two archaeal sN PylRS enzymes are fairly weakly related and fall into classes B and C, indicating they may not have a common origin.

By setting a linkage distance threshold for agglomerative clustering to 75% sequence identity (Fig. 1b) we generated eight clusters of tRNA^Pyl^ genes (Fig. 2d, Supplementary Table 1). Of these clusters, three represented the known PylRS classes N, A, and B. There were two bacterial clusters, one of which only contained a single tRNA^Pyl^. The three remaining clusters were from class C, in line with the looser interrelatedness of the members of this class.

We selected 16 pyl tRNAs – including at least one member of each tRNA cluster and pyl tRNAs with exotic structural features that are not observed in canonical class N tRNA^Pyl^ (Fig. 2e, Supplementary Note 2) – for further investigation (Fig. 2f). These 16 tRNAs included 13 pyl tRNAs which were uncharacterised in *E. coli* (six archaeal class C, and seven bacterial class S), along with three previously characterised pyl tRNAs from classes N, A and B.

To finalise our representative set of PylRS/tRNA^Pyl^ pairs for experimental characterisation, we combined each chosen tRNA^Pyl^ with the synthetase from the same organism – with the exception of A-*Alvt*RNA^Pyl^ and B-*Candidatus Methanomassiliicoccus intestinalis* (*Int*)tRNA^Pyl^, which form highly active heterologous cognate pairs with the previously reported PylRS enzymes A^Δ^- *Candidatus Methanomethylophilus sp.1R26* (1R26)Py1RS and B^Δ^-*Methanomassiliicoccus luminyensis 1* (Lum1)PylRS, respectively (Fig. 2f).^6^ We note that, of the ten inter-class relationships, only four have been even partially characterised in *E. coli* (Fig. 2g): N^+^-*Mb*PylRS is known to interact with S-*Desulfitobacterium hafniense* (*Dh*)-tRNA^Pyl^,^42^ while sets of engineered pairs from classes N, A, and B (such as N^+^-*Mm*PylRS/N-*Methanosarcina spelaei* (*Spe*)tRNA^Pyl^, A^Δ^-*1R26*PylRS/A-*Alv*tRNA^Pyl-8^, and B^Δ^-*Lum1*PylRS/B-*Int*tRNA^Pyl-17C10^), are known to be triply orthogonal to one another.^6,7^

In preparing the systems for characterisation, we observed that multiple class S PylRS genes were recalcitrant to cloning, and that even those that could be successfully cloned resulted in reduced growth when expressed in *E. coli* cells. We hypothesized that these issues might be related to their separately expressed N-terminal domain protein (PylSn), and prepared variants of each class S PylRS system with the PylSn gene removed, which abrogated the toxicity effects. These variants (which we term S^Δ^) were characterised alongside (or in place of) the wild-type enzymes (which we term S^+^).

### Active PylRS enzymes and pyl tRNAs

**Fig. 3:**
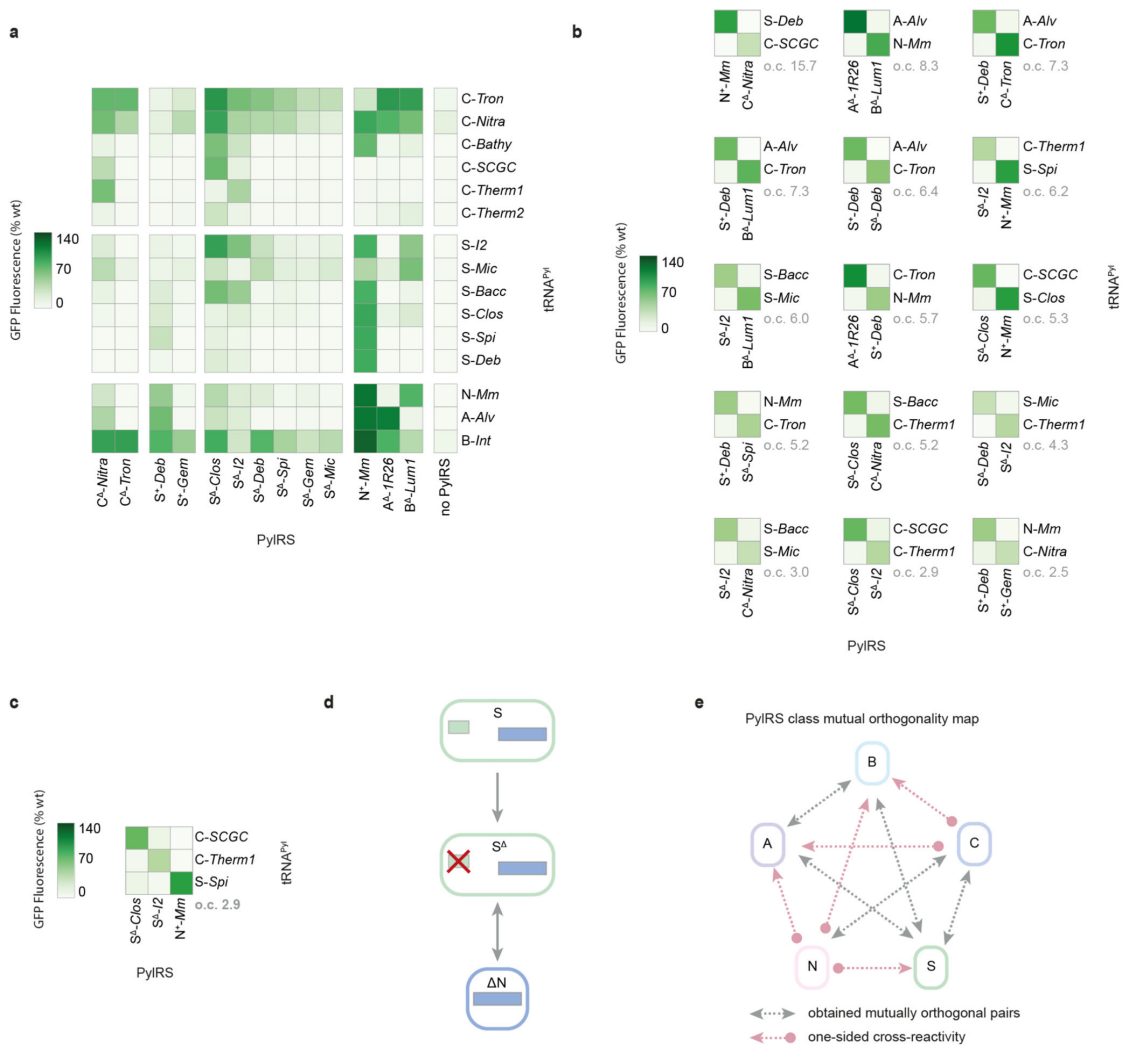
Activity mapping of candidate PylRS enzymes and pyl tRNAs, and discovery of new triply orthogonal, PylRS/tRNA^Pyl^ pairs. **a.** Heatmap displaying the activity of combinations of the selected pyl tRNAs and PylRS enzymes, measured by production of GFP(150AllocK)_His6_ from cells bearing a *GFP(150TAG)_His6_* gene in the presence of 4 mM AllocK **1**. Values are the percentage of wild-type GFP. Only PylRS enzymes and pyl tRNAs that have greater than 30% activity with at least one tRNA^Pyl^ or PylRS enzyme, respectively, are shown. Data represents the average of three biological replicates. All numerical values and bar charts including error bars showing s.d. are provided (Supplementary Table 2). **b.** Activity heatmaps of representative sets from each family of doubly orthogonal PylRS/tRNA^Pyl^ pairs obtained from the activity screen. Orthogonality coefficient (o.c) is shown in grey; the set with the highest o.c. in each family is displayed. Distinct members of a family share the same set of PylRS enzymes but use different pyl tRNAs. Data represents the average of three biological replicates. All numerical values and bar charts including error bars showing s.d. are provided (Supplementary Table 2). **c.** Activity heatmap of the set with the highest o.c. from the family of triply orthogonal PylRS/tRNA^Pyl^ pairs obtained from the activity screen. Data represents the average of three biological replicates. All numerical values and bar charts including error bars showing s.d. are provided (Supplementary Table 2). **d.** The generation of S^Δ^ PylRS variants by deletion of the N-terminal domain from class S PylRS enzymes. We considered S^Δ^ PylRS variants as engineered members of the ΔN group; their activity profiles are too diverse to be considered as a distinct class. **e.** The mutual interaction network between all five PylRS classes based on the activity between the characterised PylRS enzymes and pyl tRNAs. Mutually orthogonal pairs can be found using PylRS enzymes from: classes A and B; classes A and S; classes B and S; classes C and N; and classes C and S (double-headed grey arrows). Therefore, five out of ten possible mutually orthogonal combinations were discovered; the other five each showed one undesired cross reactivity (single-headed red arrows). These orthogonal interactions were identified without engineering to tailor PylRS:tRNA^Pyl^ interactions. For no two PylRS classes did all combinations of pairs possess two-sided cross-reactivity.

We measured the activities of each chosen PylRS enzyme with each chosen tRNA^Pyl^ *via* the production of green fluorescent protein (GFP) from a gene coding for *GFP* containing an amber codon at position 150, in the presence of the non-canonical amino acid *N*^6^-((allyloxy)carbonyl)-*L*-lysine (AllocK **1**, Extended Data Fig. 1) – a known substrate of previously characterised PylRS enzymes (Fig. 3a, Supplementary Table 2).^50^ 15 out of 16 pyl tRNAs gave rise to PylRS-dependent GFP production (at a level at least 30% of that produced from a control GFP gene without an amber stop codon, ‘wtGFP control’) in the presence of at least one PylRS enzyme (Fig. 3a). This included all class C pyl tRNAs and all but one class S tRNAPyl. In addition, 13 out of 20 PylRS enzymes led to GFP production at a level at least 30% of the wtGFP control, in the presence of at least one tRNA^Pyl^ (Supplementary Note 3). This demonstrated that most PylRS enzymes and pyl tRNAs were expressed and active in *E. coli*.

### Mutually orthogonal PylRS/tRNA^Pyl^ pairs

Next, we used our activity measurements (Fig. 3a) to determine whether any of the pyrrolysine systems we had discovered formed naturally mutually orthogonal sets (Fig. 3b,c). We first defined the criteria for mutually orthogonal pairs by reference to the interactions between them. The network of interactions between multiple aaRS/tRNA pairs may be represented as a matrix where each element *x_i,j_* is the activity of the aaRS protein of column *j* with the tRNA of row *i* (measured in this case by GFP(150AllocK)_His6_ production in the presence of aaRS_*j*_ and tRNA_*i*_). When aaRS_*i*_, denotes the cognate aaRS of tRNA_*i*_ all diagonal elements *x_i,i_* represent the paired activities we wish to maximise. All off-diagonal elements represent the cross-reactivity between a non-cognate aaRS and tRNA, which should be minimised. A diagonal interaction matrix of order *N* therefore represents a perfectly orthogonal set of *N* pairs.

In order to exclude sets of pairs with unacceptably low activity, or unacceptably high cross-reactivity, we deemed that the activity of a cognate pair should be greater than 40% of wild-type GFP production, but that each cross-reactivity (between a tRNA^Pyl^ and a PylRS enzyme belonging to different pairs) should be less than 20% of wild-type GFP production. Since pairs composed of a PylRS and tRNA^Pyl^ from different organisms can have activity equal to, or exceeding, that of the corresponding homologous pairs, we included heterologous PylRS/tRNA^Pyl^ combinations as possible cognate pairs in our search. In addition, we defined a metric, henceforth known as the ‘orthogonality coefficient’ (o.c.), as the quotient of the lowest intra-pair activity over the highest inter-pair cross-reactivity. This metric provides a quantitative measure of mutual orthogonality between a set of aaRS/tRNA pairs. Previously characterised triply orthogonal pairs (N^+^-*Mm*Py1RS/N-*Spe*tRNA^Pyl^, A^Δ^-*1R26*PylRS/A-*Alvt*RNA^Pyl-8^, B^Δ^-*Lum1*PylRS/B-*Int*tRNA^Pvl-l7C10^) used for the incorporation of three distinct non-canonical amino acids have an o.c. of approximately 5.0 (Supplementary Table 2),6 however, we reasoned that since mutual orthogonality could be improved by further engineering, a lower cut-off (o.c. > 2.5) would be more useful in initial screens.

In our initial search we considered an interaction matrix to be sufficiently orthogonal if: (i) all diagonal elements were greater than 40% of the wtGFP control, (ii) all off-diagonal elements were less than 20% of the wtGFP control, and (iii) the quotient of the smallest diagonal element over the largest off-diagonal element was greater than 2.5.

We uncovered 46 doubly orthogonal pairs (henceforth referred to as ‘doublets’); the highest doublet o.c. is 15.7. Since many doublets involve the same two PylRS enzymes, and differ only in the pyl tRNAs used, we grouped these doublets into families; members of a family share the same set of PylRS enzymes but use different pyl tRNAs. We thus obtained fifteen doublet families (Fig. 3b and Supplementary Table 2); all but one family contains a class C or class S PylRS enzyme. Similarly, we obtained two triply orthogonal pairs (or ‘triplets’), both from the same family; the highest triplet o.c. is 2.9 (Fig. 3c and Supplementary Table 2).

These families shed important insights on the PylRS/tRNA^Pyl^ activity profiles. The highly orthogonal class C pyl tRNAs – C*-Candidatus Methanohalarchaeum thermophilum 1* (Therm1)tRNA^Pyl^ and C-*Candidate division MSBL1 archaeon SCGC-AAA382A20* (*SCGC)*tRNA^Pyl^ – appear in doublets when either tRNA^Pyl^ is paired with C^Δ^-*Nitrososphaeria archaeon* (Nitra)PylRS. However, these pyl tRNAs also form a surprising inter-class doublet family when paired respectively with S^Δ^-*Desulfosporosinus sp. I2* (I2)PylRS and S^Δ^-*Clostridiales bacterium* (Clos)PylRS; this doublet forms part of a triplet family with an N+-*Mm*PylRS pair (e.g. N+-*Mm*PylRS/S-*Spirochaetales bacterium* (Spi)tRNA^Pyl^). Further doublet families involving S^+^ or S^Δ^ PylRS enzymes (e.g. with S^+^-*Deltaproteobacteria bacterium* (*Deb*)PylRS and S^Δ^-*Deb*PylRS, or S^Δ^-*Deb*PylRS and S^Δ^-*I2*PylRS) illustrate not only divergence between S^+^ PylRS enzymes and their S^Δ^ variants, but between different S^Δ^ PylRS variants. As such, we do not consider S^Δ^ PylRS enzymes as a distinct class, but rather as synthetically derived PylRS variants that expand the ΔN group (Fig. 3d).

The relationship between the five PylRS classes may itself be described on the basis of the doubly orthogonal pairs formed by representative PylRS enzymes from each class and the appropriate pyl tRNAs (which may or may not belong to the same classes). For five of these ten inter-class relationships, we obtained mutually orthogonal representative PylRS/RNA^Pyl^ pairs (Fig. 3e). For the remaining five inter-class relationships, no characterized PylRS/tRNA^Pyl^ pairs met our criteria for mutual orthogonality. Two cases for lack of mutual orthogonality between general PylRS classes R_1_ and R_2_ can be defined: (1) ‘two-sided cross-reactivity’ – for any two pairs of the form R_1_-PylRS/T_*i*_-tRNA^Pyl^ and R_2_-PylRS/T_*j*_-tRNA^Pyl^ (where T_*i*_ and T_*j*_ are arbitrary tRNA classes), both cross-reactivities R1-PylRS/T_*j*_-tRNA^Pyl^ and R_2_-PylRS/T_*i*_-tRNA^Pyl^ are too high (i.e. off-diagonal elements in the interaction matrix are greater than 20% wtGFP control or result in o.c. < 2.5); (2) ‘one-sided cross-reactivity’ – there exist pairs R_1_-PylRS/T_*i*_ -tRNA^Pyl^ and R_2_-PylRS/T_*j*_-tRNA^Pyl^ such that only one cross-reactivity R_1_-PylRS/T_*j*_-tRNA^Pyl^ or R_2_-PylRS/T_*i*_-tRNA^Pyl^ is too high (i.e. only one off-diagonal element in the interaction matrix is greater than 20% wtGFP control or results in o.c. < 2.5). Strikingly, all five non-orthogonal inter-class relationships fall into the second class. Therefore, of the 25 pairwise specificities required to make a set of quintuply orthogonal pairs (using one PylRS from each of the five classes), our computational approach was able to resolve 20 of these – all five cognate interactions and 15 out of 20 non-cognate interactions.

### Eliminating inter-class cross-reactivities

**Fig. 4:**
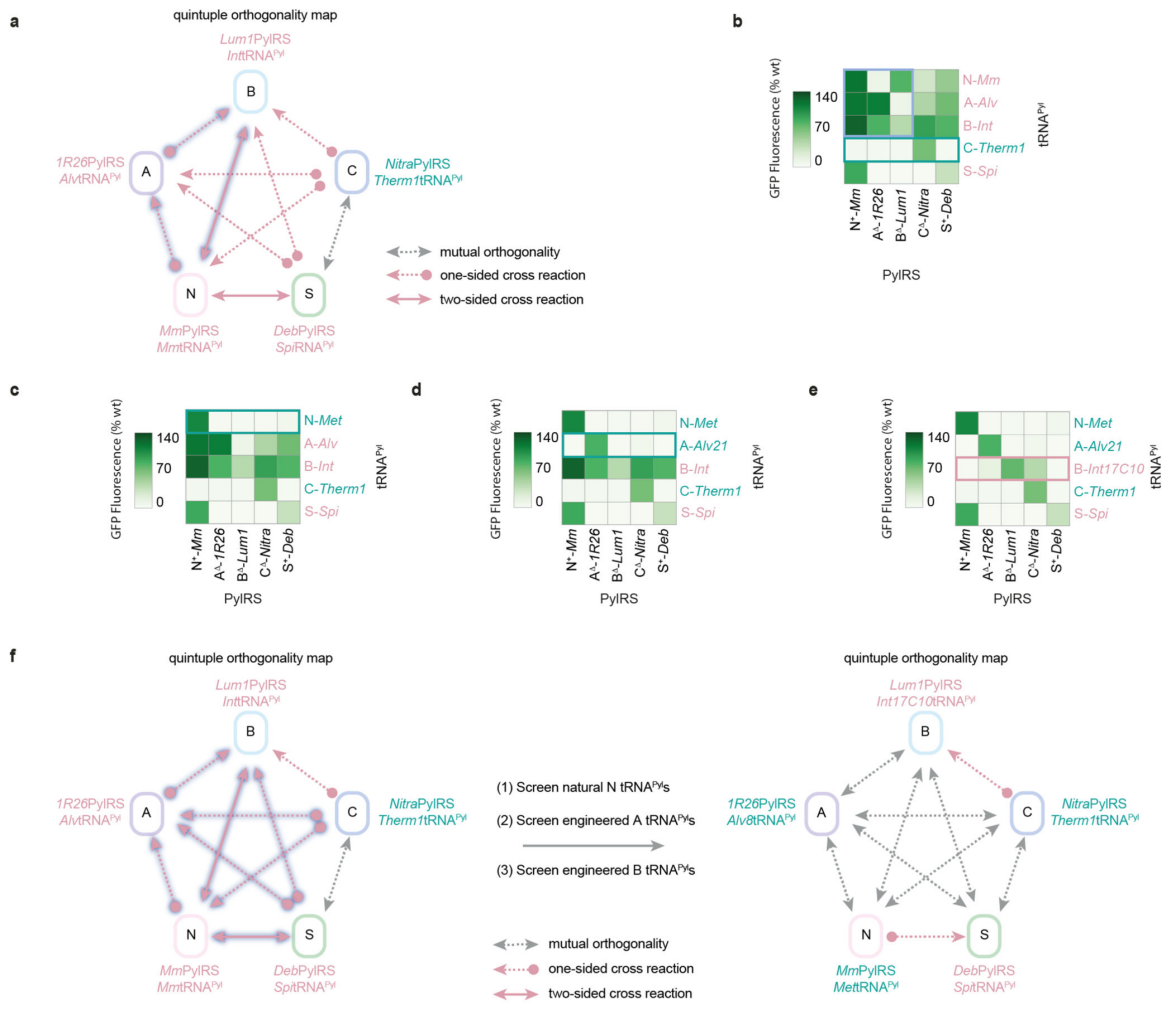
Screening of engineered pyl tRNAs permits the control of 18 out of 20 cross-reactivities between specific members of the five Pyl classes. **a.** Schematic of the interactions between five specific PylRS/tRNA^Pyl^ pairs (one from each class) that represent a logical starting point for the development of quintuply orthogonal pairs through a tRNA^Pyl^ engineering strategy. For classes N, A, and B, we chose active pairs for which the inter-class cross-reactivities (arrows highlighted in blue) have previously been controlled by tRNA^Pyl^ engineering. For class C, we chose the pair C^Δ^-*Nitra*PylRS/C-*Therm1*tRNA^Pyl^ pair, for which the tRNA^Pyl^ is naturally orthogonal to all other PylRS classes. Finally, for class S, we chose the most active intraclass PylRS/tRNAPyl pair. **b.** Activity heatmap of the set of PylRS enzymes and pyl tRNAs chosen as a basis for developing quintuply orthogonal pairs. The pyl tRNAs that require engineering or replacement to control unwanted cross-reactions are labelled in red, while the pyl tRNAs that already satisfy all necessary orthogonality requirements are labelled in green. Green box: the natural orthogonality of C-*Therm1* tRNA^Pyl^. Blue box: class interactions that have previously orthogonalized by tRNA^Pyl^ engineering and screening. Data represents the average of three biological replicates. All numerical values and bar charts including error bars showing s.d. are provided (Supplementary Table 2). **c.** Activity heatmap from **b**, updated based on the results of the class N tRNA^Pyl^ screen. N-*Met*tRNA^Pyl^, the most orthogonal tRNA from the class N tRNA^Pyl^ screen with respect to the chosen PylRS enzymes, is paired with N^+^-*Mm*PylRS. N-*Met*tRNA^Pyl^ satisfies all orthogonality requirements (green box). Data represents the average of three biological replicates. All numerical values and bar charts including error bars showing s.d. are provided (Supplementary Table 2). **d.** Activity heatmap from **c**, updated based on the results of the A-*Alvt*RNA^Pyl^ screen. A-*Alvt*RNA^Pyl-21^, the most orthogonal tRNA^Pyl^ from the class A tRNA^Pyl^ screen, is paired with A^Δ^-*1R26*PylRS. A-*Alv*tRNA^Pyl-21^ satisfies all orthogonality requirements (green box). Data represents the average of three biological replicates. All numerical values and bar charts including error bars showing s.d. are provided (Supplementary Table 2). **e.** Activity heatmap from **d**, updated based on the results of the class B tRNA^Pyl^ screen. B-*Int*tRNA^Pyl-17C10^, the most orthogonal tRNA^Pyl^ from the class B tRNA^Pyl^ screen, does not satisfy all necessary orthogonality requirements due only to cross-reactivity with C^Δ^-*Nitra*PylRS (red box). Data represents the average of three biological replicates. All numerical values and bar charts including error bars showing s.d. are provided (Supplementary Table 2). **f.** Schematic summarizing the key results of the N, A, and B tRNA^Pyl^ screens. By screening of natural and engineered pyl tRNAs, 18 out of 20 interactions – which need to be orthogonalised to generate quintuply orthogonal PylRS/tRNA^Pyl^ pairs –controlled (left diagram, arrows highlighted in blue show interactions that were successfully controlled). Three fully orthogonal pyl tRNAs (labelled in green with their cognate PylRS enzymes) were identified and for each of the remaining two pyl tRNAs (in red) only one cross reaction remains to be controlled.

As a starting point for generating a quintuply orthogonal pair, we examined the inter-class interaction matrix for five specific PylRS/tRNAPyl pairs (Fig. 4a-b). For classes N, A, and B, we used pairs N^+^-*Mm*PylRS/N-*Mm*tRNA^Pyl^, A^Δ^-*lR26*PylRS/A-*Alvt*RNAPyl, and B^Δ^-*Lum1*PylRS/B-*Int*tRNA^Pyl^, since these were the starting point for a previously reported triplet.^6^ For class C, a natural starting point is C^Δ^-*Nitra*PylRS/C-*Therm1*tRNA^Pyl^, the most active class C pair for which the tRNA^Pyl^ is orthogonal to all other PylRS classes. For class S, we simply chose the most active pairing of a wild-type class S PylRS enzyme, S^+^-*Deb*RS/S-*Spi*tRNA^Pyl^. Of the twenty possible inter-class synthetase/tRNA interactions in the matrix (off-diagonal elements), only nine are sufficiently low to meet our initial criterion for cross-reactivity (less than 20% of wild-type GFP production levels). In order to bring the other eleven interactions under this threshold, we sought to replace the tRNAs involved in undesired cross-reactions (off diagonal interactions) with more orthogonal variants, i.e. substitute the rows of the interaction matrix such that the off-diagonal elements are progressively eliminated.

To find a class N tRNA^Pyl^ with orthogonality to all other classes, we screened seven pyl tRNAs from homologous class N Pyl systems (Fig. 4c and Extended Data Fig. 2a).^6^ N-*Methanococcoides methylutens* (*Met*)tRNA^Pyl^ and N-*Methanococcoides burtonii* (*Bur*)tRNA^Pyl^ gave rise to around 10% or less activity with class A, B, C, and S PylRS enzymes while retaining over 85% of the activity of N-*Mm*tRNA^Pyl^ with N^+^-*Mm*PylRS.

To find a class A tRNA^Pyl^ with orthogonality to all other classes, we screened ten engineered variants of A-*Alv*tRNA^Pyl^ (Fig. 4d and Extended Data Fig. 2b).7 Two pyl tRNAs (A-*Alv*tRNA^Pyl-17^ and A-*Alvt* RNA^Pyl-21^) gave rise to less than 10% of wild-type GFP levels in the presence of class N, B, C, and S PylRS enzymes while retaining over 70% of activity with A^Δ^-*1R26*PylRS.

To find a class B tRNA^Pyl^ with orthogonality to all other classes, we screened seven previously reported B-*Int*tRNA^Pyl^ variants (Fig. 4e and Extended Data Fig. 2c).^6^ However, although we obtained tRNAs with orthogonality to class N, A, and S PylRS proteins, all tested pyl tRNAs gave rise to significant levels of GFP production (over 40%) in the presence of C^Δ^-*Nitra*PylRS. Since the B-*Int*tRNA^Pyl^ variants carried a range of mutations with respect to their parent tRNA^Pyl^, in both the acceptor stem and variable loop, we hypothesised that C^Δ^-*Nitra*PylRS may recognise multiple identity elements in B-*Int*tRNA^Pyl^; this suggested that the non-cognate interaction between C^Δ^-*Nitra*PylRS and B-*Int*tRNA^Pyl^ cannot easily be abrogated without also destroying recognition of B-*Int*tRNA^Pyl^ by B^Δ^-*Lum1*PylRS.

Overall, the screen led to the abrogation of a further nine inter-class PylRS/tRNA^Pyl^ interactions (Fig. 4f); this left only two undesired interactions – between the class C PylRS and class B tRNA^Pyl^, and between the class N PylRS and class S tRNA^Pyl^. Notably, three out of five tRNAs now fulfilled all orthogonality requirements. Our results demonstrate the substantial extent to which the evolutionary divergence of PylRS sequences can be exploited to generate orthogonal interactions.

### Quadruply orthogonal PylRS/tRNA^Pyl^ pairs

We investigated additional approaches to eliminate inter-class (off-diagonal) interactions for a fourth PylRS/tRNA^Pyl^ pair, and form a mutually orthogonal quadruplet.

**Fig. 5:**
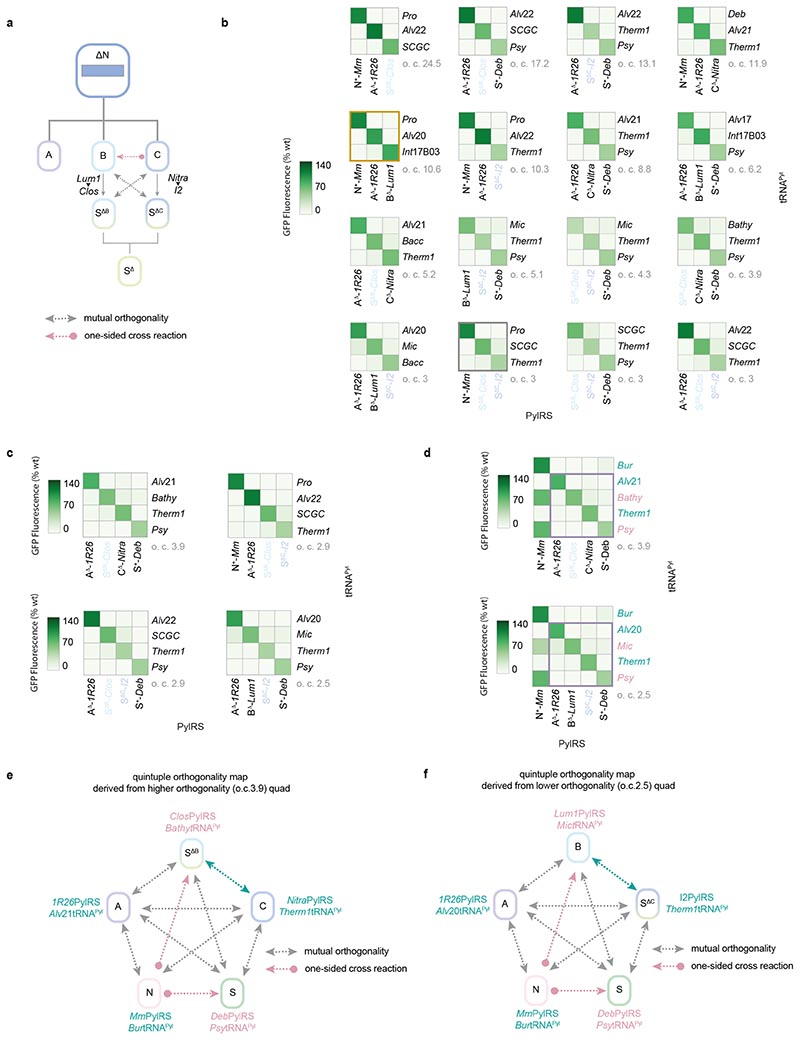
Unique activity patterns of S^Δ^ PylRS enzymes enable the development of quadruply orthogonal pairs. **a.** Schematic of the strategy of replacing a class B or C PylRS enzyme with a S^Δ^ PylRS variant to resolve the undesired cross-reactivity between class C PylRS and class B tRNA^Pyl^. The diverse activities of S^Δ^ PylRS variants mean that a different variant can be found to substitute for either class B (e.g. S^Δ^-*Clos*PylRS for B^Δ^-*Lum1*PylRS, as labelled) or class C (e.g. S^Δ^-*I2*PylRS for C^Δ^-*Nitra*PylRS, as labelled) PylRS enzymes. **b.** Activity heatmaps of the highest o.c. sets from each family of triply orthogonal PylRS/tRNA^Pyl^ pairs obtained following the results of the N, A, and B tRNA^Pyl^ screens. The substitution of a B or C class PylRS with different S^Δ^ PylRS variants (labelled in two shades of blue) allows the generation of many of the new families. Gold box: representative set from the previously reported triply orthogonal N^+^-*Mm*PylRS, A^Δ^-*1R26*PylRS, B^Δ^-*Lum1*PylRS family. Silver box: representative set from the only triply orthogonal family found prior to the N, A, and B tRNA^Pyl^ screens. Orthogonality coefficient, o.c. is shown in grey. Distinct members of a family share the same set of PylRS enzymes but use different pyl tRNAs. Data represents the average of three biological replicates. All numerical values and bar charts including error bars showing s.d. are provided (Supplementary Table 2). **c.** Activity heatmaps of the highest o.c. sets from each family of quadruply orthogonal PylRS/tRNA^Pyl^ pairs obtained following the results of the class N, A, and B tRNA^Pyl^ screens. The substitution of a class B or C PylRS with different S^Δ^ PylRS variants (labelled in two shades of blue) allows the generation of all such families. Data represents the average of three biological replicates. All numerical values and bar charts including error bars showing s.d. are provided (Supplementary Table 2). **d**. Activity heatmaps of the two quadruplet families with a single S^Δ^ PylRS variant substituting for a class B or class C PylRS, shown along with the most orthogonal fifth pair from the final class (class N). The pyl tRNAs that require engineering or replacement to minimize unwanted cross-reactions are labelled in red, while the pyl tRNAs that already satisfy all necessary orthogonality requirements are labelled in green. Data represents the average of three biological replicates. All numerical values and bar charts including error bars showing s.d. are provided (Supplementary Table 2). **e.** Schematic of the interactions between the quadruply orthogonal set with the highest o.c. (class B PylRS substituted by S^ΔB^, o.c. 3.9) and the most orthogonal fifth pair from the final class (class N). **f.** Schematic of the interactions between the quadruply orthogonal set with the lowest o.c. (class C PylRS substituted by S^ΔC^, o.c. 2.5) and the most orthogonal fifth pair from the final class (class N).

As noted above, the engineered S^Δ^PylRS variants belong to the expanded ΔN group (Fig. 3d). The activities of different S^Δ^ variants resemble the activities of different ΔN classes; for instance S^Δ^-*I2*PylRS is active with C-*Therm1*tRNA^Pyl^ (Fig. 3a; much like C^Δ^-*Nitra*PylRS), and S^Δ^-*Clos*PylRS is active with engineered B-*Int*tRNA^Pyl^ variants (Extended Data Fig. 2c; much like B^Δ^-*Lum1*PylRS). We therefore speculated that substitution of the class B or C pair with a pair containing a S^Δ^ PylRS supplying a desired B-like or C-like activity might resolve the issue of cross-reactivity between class C PylRS enzymes and class B pyl tRNAs (Fig. 5a). We refer to these substituting PylRS variants as S^ΔB^ or S^ΔC^, respectively.

By allowing the replacement of class B or C PylRS enzymes with S^Δ^ PylRS variants, we obtained a total of 946 doublets in 25 families, 1425 triplets in 16 families, and – crucially – 96 quadruplets in four families (Fig. 5b-c and Supplementary Table 2). Notably, the highest triplet o.c. is 24.5 – approximately five times higher than a previously reported triplet used for the incorporation of three distinct non-canonical amino acids (N^+^-*Mm*PylRS/N-*Spet*RNA^Pyl^, A^Δ^-*1R26*PylRS/A-*Alvt*RNA^Pyl-8^, B^Δ^-*Lum1*PylRS/B-*Int*tRNA^Pyl-17C10^, Supplementary Table 2), and approximately two times higher than the highest o.c. triplet from the previously reported N^+^-*Mm*PylRS, A^Δ^-*1R26*PylRS, B^Δ^-*Lum1*PylRS family.^6^ Most triplet and all quadruplet families involve substitution of the class B and/or class C pair with pairs containing S^Δ^ PylRS variants.

To understand how far the S^Δ^ PylRS substitution strategy had advanced the development of quintuply orthogonal pairs, we considered the quadruplets in the context of our inter-class interaction network (Fig. 5d-f and Extended Data Fig. 3). For the [A, S ^ΔB^, C, S] (o.c. 3.9) and [A, B, S^ΔC^, S] (o.c. 2.5) quadruplet families formed with a single S^Δ^ PylRS variant substituting for a class B or class C PylRS respectively, the cross-reactivity between classes B and C (Fig. 4e) had effectively been replaced by cross-reactivity between class N and S^ΔB^ or B (Fig. 5d-f). This results in the generation of a diagonal submatrix of order 4 in the overall interaction matrix, and thus the quadruplets. However, including cross-reactivity between classes N (N^+^-*Mm*PylRS) and S (any tRNA^Pyl^ paired with S^+^-*Deb*PylRS), two cross-reactivities still remained to be eliminated. Meanwhile, for the [N, A, S^ΔB^, S^ΔC^] and [A, S^ΔB^, S^ΔC^, S] quadruplets (both o.c. 2.9) formed *via* substitution of both class B and class C PylRS enzymes with S^Δ^ PylRS variants, one main cross-reactivity (between N^+^-*Mm*PylRS and any tRNA^Pyl^ paired with S^+^-*Deb*PylRS) persisted (Extended Data Fig. 3). However, there also remained some residual cross-reaction between the class S^ΔB^ and S^ΔC^ pairs which would restrict the o.c. of any potential quintuplets. We hypothesised that a general solution to all of these cross-reactivity problems would be further engineering of the pyl tRNAs paired with class B/S^ΔB^ and class S PylRS enzymes.

### Quintuply orthogonal PylRS/tRNA^Pyl^ pairs

**Fig. 6:**
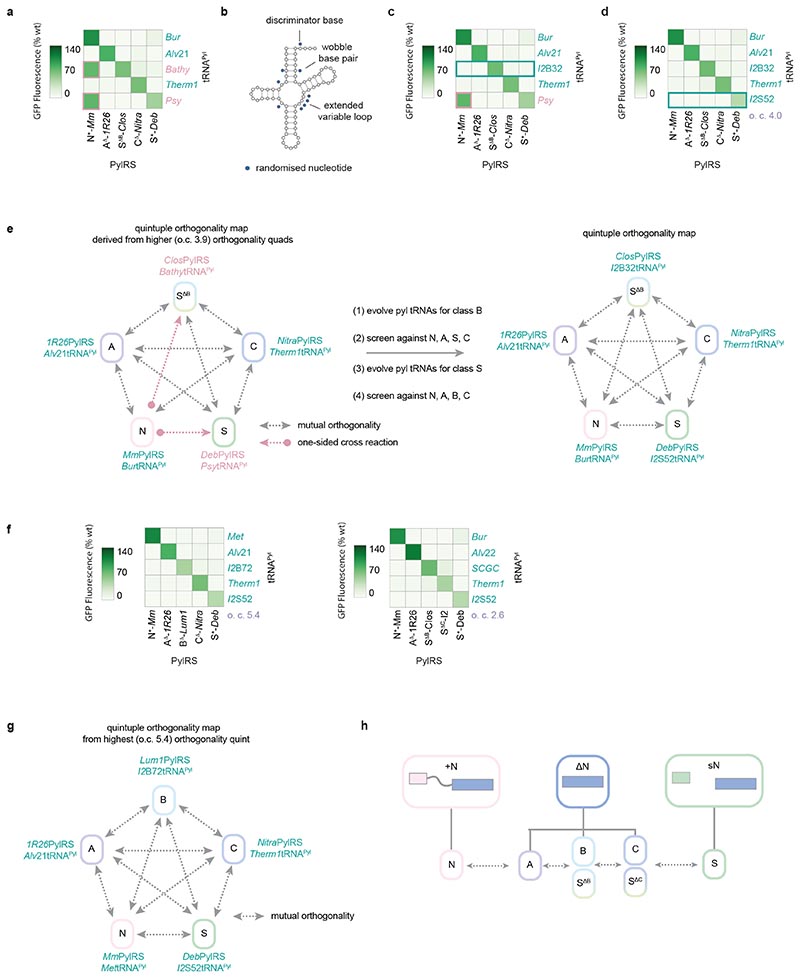
Quintuply orthogonal PylRS/tRNA^Pyl^ pairs via directed evolution. **a.** Activity heatmap combining the quadruplet with the highest o.c. and the most orthogonal fifth pair from the final class. Cross-reactivities are boxed in red; pyl tRNAs to be replaced with quintuply orthogonal evolved variants are labelled in red. **b.** The library used for evolution of quintuply orthogonal pyl tRNAs from the S-*I2*tRNA^Pyl^ scaffold. The cloverleaf structure of S-*I2*tRNA^Pyl^ is shown. **c.** Activity heatmap from **a**, updated based on the results of the directed evolution of pyl tRNAs specific to class B (or S^ΔB^). S-*I2*tRNA^Pyl-B3^2, the most orthogonal tRNA from the directed evolution, satisfies all necessary orthogonality requirements (green box). As a result, the quintuply orthogonal S^ΔB^-*Clos*PylRS/S-*I2*tRNA^Pyl-B32^ pair substitutes effectively for class B and requires no further engineering. Data represents the average of three biological replicates. All numerical values and bar charts including error bars showing s.d. are provided (Supplementary Table 2). **d.** Activity heatmap from **c**, updated based on the results of the directed evolution of a pyl tRNA specific to class S. S-*I2*tRNA^Pyl-S52^ satisfies all necessary orthogonality requirements (green box). As a result, the S^+^-*Deb*PylRS/S-*I2*tRNA^Pyl-S52^ pair requires no further engineering, and completes a quintuply orthogonal set of pairs (o.c. 4.0). Data represents the average of three biological replicates. All numerical values and bar charts including error bars showing s.d. are provided (Supplementary Table 2). **e.** Schematic of the overall tRNA^Pyl^ evolution strategy and resulting pairs. The cross-reactions between class N and class B (or its S^Δ^ equivalent), and between class N and class S are successively destroyed to yield a set of five pairs where all twenty cross-reactions are minimized. **f.** Activity heatmaps from two families of quintuply orthogonal pairs that incorporate the evolved pyl tRNAs; the quintuplets with the highest o.c.. Data represents the average of three biological replicates. All numerical values and bar charts including error bars showing s.d. are provided (Supplementary Table 2). **g.** Schematic of the interactions within the quintuply orthogonal set of pairs with the highest o.c. (5.4), which is formed with one PylRS from each class. **h.** Schematic highlighting the successful division of pyrrolysine systems into five mutually orthogonal functional classes: N, A, B or S^ΔB^, C or S^ΔC^, and S.

We aimed to: (1) discover a tRNA^Pyl^ that functions with a class B PylRS enzyme (or S^ΔB^ PylRS variant) but is orthogonal to all other PylRS classes, (2) discover a tRNA^Pyl^ that functions with a class S PylRS but is orthogonal to all other PylRS classes, and (3) replace the pyl tRNAs that pair with S^ΔB^-*Clos*PylRS and S^+^-*Deb*PylRS with our new pyl tRNAs in the highest o.c. quadruplet. We anticipated that this would mitigate undesired cross-reactivity with a fifth pair and thereby enable the generation of a quintuply orthogonal set of pairs (Fig. 6a).

We noted that engineered B-tRNA^Pyl^ variants were active with C^Δ^-*Nitra*PylRS (Extended Data Fig. 2c), and postulated that a different parent tRNA^Pyl^, such as one from a bacterial class S system, might provide a better starting point for the discovery of a class B- or S^ΔB^-specific tRNA^Pyl^ variant with orthogonality towards C^Δ^-*Nitra*PylRS (and the other PylRS enzymes, as necessary), via directed evolution.

We chose S-*I2*tRNA^Pyl^, a bacterial tRNA, as a starting point for directed evolution; this tRNA has exceptionally high activity with S^ΔB^-*Clos*PylRS (giving rise to 93% of wild-type GFP levels), yet fairly modest cross-reactivity with C^Δ^-*Nitra*PylRS (23% of wild-type GFP levels). In fact, S-*I2*tRNA^Pyl^ is most cross-reactive with N+-*Mm*PylRS (68% of wild-type GFP levels); accordingly, we focussed our efforts on abrogating S-*I2*tRNA^Pyl^ recognition by N+-*Mm*PylRS.

Previous work demonstrated that N^+^-*Mm*PylRS can be much more sensitive to expansions in the short variable loop of tRNA^Pyl^ than ΔN PylRS enzymes from classes A^7^ and B^6^ (Supplementary Table 2). Since S^ΔB^-*Clos*PylRS is an artificial ΔN class PylRS, we hypothesised that it may tolerate insertions into the variable loop of S-*I2*tRNA^Pyl^, and synthesised a library of S-*I2*tRNA^Pyl^ mutants in which the variable loop was expanded to four nucleotides (Fig. 6b). Positions 8 and 22, which could make important tertiary contacts with the variable loop, were also randomised. In addition, to widen the diversity of activity profiles within the library, three bases thought to be generally important to the recognition of S-*I2*tRNA^Pyl^ were also randomised; namely, position 69 (the discriminator base, an important identity element for PylRS), and positions 5 and 64. The latter two form a wobble base pair in the S-*I2*tRNA^Pyl^ acceptor stem; such pairs are known to distort RNA helices and thereby dictate aminoacyl-tRNA synthetase recognition.^51^

We selected S-*I2*tRNA^Pyl^ mutants that allowed cells also expressing S^ΔB^-*Clos*PylRS to grow on 100 μg mL^-1^ chloramphenicol in the presence of AllocK **1**, by enabling production of protein from a gene coding for chloramphenicol acetyl transferase containing an amber codon at position 111. We then performed successive screens on the selected S-*I2*tRNA^Pyl^ variants to identify pyl tRNAs that have minimal cross-reactivity with N^+^-*Mm*PylRS, A^Δ^-*1R26*PylRS, C^Δ^-*Nitra*PylRS, and S^+^-*Deb*PylRS. Cells harbouring *GFP(150TAG)_His6_*, an S-*I2*tRNA^Pyl^ variant, and one of N^+^-*Mm*PylRS, A^Δ^-*1R26*PylRS, C^Δ^-*Nitra*PylRS, or S^+^-*Deb*PylRS were provided with AllocK and screened for the absence of GFP expression. These screens revealed three library members, S-*I2*tRNA^Pyl-B8^, S-*I2*tRNA^Pyl-B32^, and S-*I2*tRNA^Pyl-B72^, which remain highly active with S^ΔB^-*Clos*PylRS but have little cross-reactivity with N^+^-*Mm*PylRS, A^Δ^-*1R26*PylRS, C^Δ^-*Nitra*PylRS, and S^+^-*Deb*PylRS (Fig. 6c and Extended Data Fig. 4a). In particular, S-*I2*tRNA^Pyl-B32^ and S-*I2*tRNA^Pyl-B72^ both retain over 75% of the activity of their parent S-*I2*tRNA^Pyl^ with S^ΔB^-*Clos*PylRS but have over 20-fold lower activity than their parent tRNA with N^+^-*Mm*PylRS and around three-fold lower activity than their parent with C^Δ^-*Nitra*PylRS.

While performing screens on S-*I2*tRNA^Pyl^ mutants, we observed that – despite their expanded variable loops – some S-*I2*tRNA^Pyl^ mutants have increased activity with S^+^-*Deb*PylRS. We hypothesised that the N-terminal domain protein in the split S^+^-*Deb*PylRS enzyme may have different recognition of variable loop nucleotides from N^+^ PylRS. Therefore, to discover a tRNA^Pyl^ that pairs with a class S PylRS that is orthogonal to all other PylRS classes used in a quintuplet, we selected S-*I2*tRNA^Pyl^ expanded variable loop mutant library members that are selectively aminoacylated by S^+^-*Deb*PylRS.

We performed a positive selection for S-*I2*tRNA^Pyl^ mutants that are active with S^+^-*Deb*PylRS, followed by screening to minimize cross-reactivity with N^+^-*Mm*PylRS, A^Δ^-*1R26*PylRS, S^ΔB^-*Clos*PylRS, and C^Δ^-*Nitra*PylRS. These screens identified one library member, S-*I2*tRNA^Pyl-S52^, which is active with S^+^-*Deb*PylRS (43% of wild-type GFP production) but possesses very low activity with S^Δ^-*Clos*PylRS, N^+^-*Mm*PylRS, A^Δ^-*1R26*PylRS, and C^Δ^-*Nitra*PylRS (all less than 2% of wild-type GFP production) (Fig. 6d and Extended Data Fig. 4b). When compared with the wild-type (S-*I2*tRNA^Pyl^), S-*I2*tRNA^Pyl-S52^ is over three-fold more active with S^+^-*Deb*PylRS and 120-fold less active with S^ΔB^-*Clos*PylRS.

By substituting our S-*I2*tRNA^Pyl^ mutants into the quadruplet with the highest o.c. (from the [A, S^ΔB^, C, S] family), we minimized cross-reactivities with N^+^-*Mm*PylRS. This enabled us to combine the updated quadruplets with a fifth orthogonal pair from class N (for example N^+^-*Mm*PylRS/N-*Burt*RNA^Pyl^) to generate a family of mutually orthogonal quintuplets with o.c. values of up to 4.0 (Fig. 6d). Thus, by mutation, selection and screening from a single tRNA scaffold we eliminated the two final cross-reactivities (Fig. 6e). Despite their very different activities, S-*I2*tRNA^Pyl-B32^ and S-*I2*tRNA^Pyl-S52^ differ by only four nucleotides; this demonstrates the power of synthetically engineering identity elements to control tRNA recognition by aminoacyl-tRNA synthetases.

Because S-*I2*tRNA^Pyl-S^52 is also orthogonal to both B^Δ^-*Lum1*PylRS and S^ΔC^-*I2*PylRS, we obtained two additional quintuplet families, by substitution of S^ΔB^-*Clos*PylRS with B^Δ^-*Lum1*PylRS, or C^Δ^-*Nitra*PylRS with S^ΔC^-*I2*PylRS. Overall, using our original o.c. threshold of 2.5, we obtained 1136 doublets in 27 families (highest o.c. 72.0), 2359 triplets in 26 families (highest o.c. 39.6), 919 quadruplets in 14 families (highest o.c. 7.2), and 90 quintuplets in 3 families (highest o.c. 5.4). Upon increasing the o.c. threshold to 5.0 – comparable to the o.c. value for PylRS/tRNA^Pyl^ pairs previously used for incorporating three distinct non-canonical amino acids – we obtained 924 doublets in 22 families, 1324 triplets in 18 families, 128 quadruplets in 7 families, and 8 quintuplets in 1 family (Fig. 6f, Extended Data Fig. 4c, Supplementary Fig. 4, Supplementary Table 2). The quintuplets with the highest o.c. values have the following composition: N^+^-*Mm*PylRS with a (non-cognate) natural class N tRNA^Pyl^; A^Δ^-*1R26*PylRS with an engineered A-*Alvt*RNA^Pyl^ variant; B^Δ^-*Lum1*PylRS with an engineered S-*I2*tRNA^Pyl^ variant; C^Δ^-*Nitra*PylRS with a (non-cognate) natural class C tRNA^Pyl^; and S^+^-*Deb*PylRS with an engineered S-*I2*tRNA^Pyl^ variant (Fig. 6g). We characterized the amber suppression efficiency and accuracy of each quintuply orthogonal PylRS/tRNA^Pyl^ pair in the most orthogonal quintuplet by producing GFP150AllocK_His6_ and Ub11AllocK_His6_ from *GFP150TAG_His6_* or *Ub11TAG_His6_* respectively, and measuring protein titres as well as MS spectra (Supplementary Fig. 5 to 10 and Supplementary Table 4 and 5). Our results demonstrate the successful and unprecedented division of homologous PylRS/tRNA^Pyl^ systems into five classes that are mutually orthogonal in their aminoacylation specificity (Fig. 6h).

## Discussion

We have defined sequence identity threshold criteria to effectively search genomic data for PylRS and tRNA^Pyl^ orthogonality. By applying these thresholds to generate sequence clusters we have computationally searched PylRS sequences for multiply orthogonal systems. Using this approach we have identified orthogonal systems from hundreds of PylRS systems spanning all PylRS groups. By combining our computational approach with directed evolution and engineering we have generated the first quadruply and quintuply orthogonal PylRS systems. These advances, along with strategies for generating codons that can be used to encode non-canonical monomers, may facilitate the synthesis of proteins containing an increasing number of ncAAs, and the encoded cellular synthesis of more diverse polymers and macrocycles.^2,3,18,19,21–26^ Moreover, mining the data generated through our approach may provide further insight into the sequence requirements for mutually orthogonal systems and enable the creation of more refined rules for predicting orthogonality.

## Methods

### Identification of PylRS sequences

We identified PylRS sequences by performing a BLAST search against the NCBI non-redundant protein sequence database using the A^Δ^-*Alv*PylRS protein as the query sequence, filtering for expected values below 1 x 10^-30^. Partial protein sequences and sequences from synthetic constructs were removed by manual inspection, and we ultimately obtained 351 PylRS sequences. We collected these sequences into a database, where each PylRS sequence was assigned a unique identifier based on the organism name and the NCBI Accession ID. We aligned the obtained PylRS sequences using *Clustal Omega*^52^ and extracted sequences corresponding to the C terminal domain (CTD) of the PylRS enzymes, with reference to the known annotation of the CTD in N^+^-*Mm*PylRS^6^.

### Identification of tRNA^Pyl^ sequences

Using the NCBI Nucleotide database, we obtained all available nucleotide sequences of the host genome or metagenomic read containing each of our identified PylRS sequences. We ran the tRNA detection program *ARAGORN49(version* 1.2.38) on each nucleotide sequence, allowing for introns of up to 100 nucleobases and scoring thresholds at 90% of default levels. Discovered pyl tRNAs were added to the PylRS sequence database. For the PylRS sequence with identifier *Marc.6481* (isolated from candidate division *MSBL1* archaeon *SCGC-AAA382A20*, subsequently referred to as C^Δ^-*SCGC*PylRS), no corresponding tRNA could be found by *ARAGORN*, but a putative sequence had been previously reported^53^ and was therefore added to the database. Meanwhile, PylRS sequences with identifiers *Mthe.7552* and *Mthe.9096* were found to originate from the same organism (*Candidatus Methanohalarchaeum thermophilum*), but *ARAGORN* only found a corresponding tRNA^Pyl^ proximate to one of the PylRS sequences (*Mthe.7552*, subsequently referred to as C^Δ^-*Therm1*PylRS). Manual searching of the nucleotide sequence revealed a second tRNA^Pyl^ proximate to the other PylRS sequence (*Mthe.9096*, subsequently referred to as C^Δ^-*Therm2*PylRS); this was also added to the database. During preparation of this manuscript these two pyl tRNAs were independently reported.^46^ After manual curation to remove pseudogenes, we obtained pyl tRNAs for 284 PylRS genes.

### Analysis of previously characterised PylRS/tRNA^Pyl^ pairs

From our database of PylRS and tRNA^Pyl^ sequences, we obtained the sequences of class A and B PylRS and tRNA^Pyl^ sequences that we had previously characterized experimentally.^6^ A matrix of pairwise sequence percentage identities for all pairs of PylRS sequences and all pairs of tRNA^Pyl^ was then calculated using *python* (version 3.9.7).^54^ For PylRS sequences, percentage identities were calculated from the multiple sequence alignment of C terminal domains. For tRNA^Pyl^ sequences, percentage identities were calculated from a manually performed multiple sequence alignment that was based on secondary structure predictions from *ARAGORN* and *RNAfold* .^55,56^

We considered the experimental activity data previously reported for these sequences, namely the level of GFP production from a GFP gene containing an in-frame amber codon at position 150 (*GFP150TAG_His6_*) obtained in the presence of each combination of PylRS enzyme, and tRNA^Pyl^_CUA_, as well as 8 mM *N*^ε^-Boc-*L*-lysine (subtracted by the level of GFP production in the presence of only GFP gene and tRNA^Pyl^_CUA_). For each combination of PylRS enzyme and tRNA^Pyl^_CUA_, we plotted this activity against the percentage sequence identity of the PylRS sequence with the sequence of the PylRS from the same organism as the tRNA^Pyl^_CUA_. Similarly, we plotted the activity for each combination of PylRS enzyme and tRNA^Pyl^_CUA_ against the percentage identity of the tRNA^Pyl^_CUA_ with the sequence of the tRNA^Pyl^_CUA_ from the same organism as the PylRS enzyme.

### Clustering of PylRS C terminal domain sequences

Using *python* (version 3.9.7), we calculated a matrix of percentage identities from the multiple sequence alignment of C terminal domains for all pairs of PylRS sequences in our database. We then used this matrix to perform unweighted average linkage agglomerative hierarchical clustering (UPGMA) of aligned PylRS CTD sequences with a cluster merging threshold of 55% sequence identity, using the *biopython* (version 1.79) and the *scikit-learn* (version 1.0.1) *python libraries*.^54,57^

### Alignment and clustering of tRNA^Pyl^ sequences

For each PylRS cluster, we chose a representative PylRS sequence for which a corresponding tRNA^Pyl^ sequence could be found. For two of the 37 PylRS clusters, no tRNA^Pyl^ sequence was found; these were excluded from further analysis. For the 35 obtained pyl tRNAs, we performed a manual multiple sequence alignment that was based on secondary structure predictions from *ARAGORN* and *RNAfold*. This multiple sequence alignment was then used to calculate a matrix of percentage identities for all pairs of chosen tRNA^Pyl^, using the *biopython* (version 1.79) and *scikit-learn* (version 1.0.1) *python* libraries. We then used this matrix to perform unweighted average linkage agglomerative hierarchical clustering (UPGMA) of aligned chosen tRNA^Pyl^ sequences with a cluster merging threshold of 75% sequence identity.

### DNA constructs

PylRS and tRNA^Pyl^ genes were synthesized by IDT as gBlock double-stranded DNA fragments. We cloned all new pyl tRNAs into a minimal pMB1 backbone under an *lpp* promoter. Previously reported pyl tRNAs were used in the same format. Certain tRNAs differed from the canonical sequence at the anticodon loop; these positions were mutated to the consensus bases found in *E. coli* to improve the efficiency of the tRNAs in *E. coli* translation as has been previously described.^6^ PylRS sequences were cloned into a p15A backbone under a *glnS* promoter. For each new PylRS sequence, a 5’ untranslated region was generated using the online tool *De Novo DNA*^58–63^ predicted to maximise translation initiation efficiency (see Supplementary Table 3) and inserted between the +1 site of the *glnS* promoter and the start codon of the gene. For class S PylRS enzymes, polycistronic operons consisting of the separately expressed N- and C-terminal domains were constructed using intergenic regions predicted by *De Novo DNA*. The optimal arrangement of the two domains was chosen by maximizing predicted translation initiation rates. *N^+^Mm*PylRS, A^Δ^-*Alv*PylRS, and B ^Δ^-*Lum1*PylRS were used in similar p15A constructs containing C-terminal tags as previously described.^6^ The p15A vectors also encoded a *chloramphenicol acetyltransferase* (CAT) gene with an amber codon at position 111 under a constitutive *cat* promoter and a GFP gene with an amber codon at position 150 under an *L*-arabinose inducible *pBAD* promoter.

### Measuring the activity and specificity of PylRS/tRN^Pyl^_CUA_ pairs

To measure the activity of the PylRS/tRNA^Pyl^_CUA_ pairs we transformed 0.4 μL of pMB1 plasmid encoding a tRNA^Pyl^_CUA_ gene into 4-10 μL *E.coli* DH10B chemically competent cells bearing a p15A plasmid encoding a *PylRS* gene, a *CAT111TAG* gene, as well as a *GFP150TAG_His6_* gene. We recovered the transformed cells for approximately 1h at 37°C and 75Or.p.m. in 180 μL of SOC medium (Super optimal broth with catabolite repression) in a 96 well *Costar* microtitre plate format. We then used 40 μL of the rescued cells to inoculate 760 μL of selective 2xYT-st (2xYT medium containing 75 μg mL^-1^ spectinomycin and 12.5 μg mL^-1^ tetracycline) medium in a 1.2 mL 96 well plate format and the cultures were grown overnight at 37°C and 750 r.p.m. After a minimum of 16 h, 40 μL of the overnight cultures were used to inoculate 760 μL of 2xYT-st medium, containing 0.05% *L*-arabinose and 4 mM *N^ε^*-Alloc-*L*-lysine (AllocK), in a 1.2 mL 96 well plate format. Cells were grown for 18-24 h at 37°C and 750 r.p.m. Ultimately, 100 μL of each culture was transferred into 96 well flat bottom *Costar* plates and fluorescence and optical density (OD) were measured using a *PHERAstar* FS plate reader. Measured GFP/OD600 values were normalised by the GFP/OD_600_ value of cells expressing GFP from a *GFP150Asn_His6_* gene (referred to as ‘wtGFP control’).

### Identification of sets of mutually orthogonal PylRS/tRNA^Pyl^ pairs

Using *python* (version 3.9.7), we identified sets of mutually orthogonal PylRS/tRNA^Pyl^ pairs based on the GFP activity data. For any given set of PylRS/tRNA^Pyl^ pairs, the quotient of the lowest intra-pair activity over the highest inter-pair cross reactivity was defined as the orthogonality coefficient, o.c.. Sets of pairs were considered mutually orthogonal if the lowest intra-pair activity was greater than 40% of the wtGFP control, the highest inter-pair cross-reactivity was less than 20% of the wtGFP control, and the o.c. was higher than 2.5. We grouped mutually orthogonal sets together into families if they involved the same PylRS enzymes.

### S-*I2*tRNA^Pyl^_CUA_ library generation

The library of S-*I2*tRNA^Pyl^_CUA_ with randomized nucleotides was constructed by Golden Gate cloning into a pMB1 vector using PCR primers as listed in Supplementary Table 3, a Q5 DNA polymerase, a Bbs1-HF restriction enzyme, and a T4 DNA ligase (all enzymes were purchased *from New England Biolabs* (*NEB*)). The library was transformed into electrocompetent *E.coli* DH10B cells with a transformation efficiency of more than 1x108 colony forming units.

### Selection and screening to identify orthogonal *S-I2*tRNA^Pyl^_CUA_ hits

The S-*I2*tRNA^Pyl^_CUA_ library was transformed into electrocompetent *E.coli* DH10B cells bearing a p15A plasmid encoding *CAT(111TAG)*, *GFP(150TAG)_His6_* and either S^Δ^-*Clos*PylRS or S^+^-*Deb*PylRS. Cells were recovered for one hour in 1 mL SOC at 37°C 220 r.p.m. supplemented with AllocK and plated onto LB agar plates containing 4 mM AllocK, 75 μgmL^-1^ spectinomycin, 12.5 μgmL^-1^ tetracycline and 100 μgmL^-1^ chloramphenicol. The plates were incubated at 37°C for 18-24 h. After incubation a combined total of 576 colonies from either selection (colonies grown in presence of S^Δ^-*Clos* PylRS or S^+^-*Deb*PylRS, respectively) were picked into 500 μL 2xYT-st and the colonies were grown over night at 750 r.p.m. and 37°C. 40 μL of the overnight culture were then given into 760 μL 2xYT-st containing 0.05% *L*-arabinose in presence and absence of 4 mM AllocK. Plasmids from clones which were selectively fluorescent in presence of AllocK were extracted (DNA miniprep from *Qiagen*), digested with NcoI restriction enzyme and T5 exonuclease (both from *NEB*) and retransformed into chemically competent cells bearing a p15A plasmid encoding *CAT(111TAG), GFP(150TAG)_His6_* and one of the following PylRS genes – N^+^-*Mm*PylRS, S^Δ^-*Clos*PylRS, C^Δ^-*Nitra*PylRS, A^Δ^-*1R26*PylRS, or S^+^-*Deb*PylRS. Plasmids of clones which fulfilled the orthogonality requirements were isolated and sequenced.

### Quantifying GFP150AllocK_His6_ and Ub11AllocK_His6_ protein production yields with quintuply orthogonal PylRS/tRNA^Pyl^_CUA_ pairs

To measure the protein yield for single ncAA incorporations with the quintuply orthogonal PylRS/tRNA^Pyl^_CUA_ pairs from the set with the highest o.c., we co-transformed the pMB1 plasmid (encoding a *tRNA^Pyl^CUA* gene) and the p15A plasmid (encoding a *PylRS* gene, a *CAT111TAG* gene, as well as a *GFP150TAG_His6_*, or *Ub11TAG_His6_*gene) into competent *E.coli* DH10B by electroporation. As controls we also co-transformed *GFP_His_* or *Ub11TCA_His6_* together with *Alvt*RNA^Pyl-21^_CUA_ and *Mm*PylRS in the same plasmid set-up.

We recovered the transformed cells for approximately 1 h at 37°C and 220 r.p.m. in 600 μL of SOC medium (Super optimal broth with catabolite repression). We used 160 μL of the rescued cells to inoculate 5 mL of selective 2xYT-st (2xYT medium containing 75 μg mL^-1^ spectinomycin and 12.5 μg mL^-1^ tetracycline) medium in a 50 mL glass tubes and the cultures were grown overnight at 37°C and 220 r.p.m in a shaking incubator. After a minimum of 16 h, 140 μL of the overnight cultures were used to inoculate 5 mL of 2xYT-st medium, containing 0.05% *L* -arabinose and 4 mM *N*-Alloc-*L*-lysine (AllocK), in a 50 mL glass tube. Cells were grown for 16-18 h at 37°C and 220 r.p.m. Cells were spun down, aspirated and the cell pellets were frozen at -20 °C for a minimum of 1 h.

The pellets were resuspended in 800 μL BugBuster® Protein Extraction Reagent containing cOmplete™ protease inhibitor and lysed for one hour with head-over-tail rotation. Lysed cells were spun down and the supernatant incubated for 1-16 h at 4 °C with 160 μL NiNTA agarose beads. The beads were washed five times with 800 μL 25 mM imidazole in PBS at pH 8.5 and the proteins were eluted five times with 160 μL 250 mM imidazole in PBS pH 8.5 (for GFP samples), or five times with 100 μL 250 mM imidazole in PBS pH 8.5 (for Ub samples). Protein concentrations for GFP were measured by quantifying the absorption at 280 nm. Protein concentrations for ubiquitin were measured using Pierce™ BCA Protein Assay Kit from *Thermo Fisher* following the manufacturers protocol.

### Electrospray ionization mass spectrometry

Denatured protein samples (~10 μM) were subjected to liquid chromatography-mass spectrometry analysis. Briefly, proteins were separated on a C4 BEH 1.7 μm, 1.0 × 100 mm ultraperformance liquid chromatography column (Waters) using a modified nanoAcquity (Waters) to deliver a flow of approximately 50 μl min^-1^. The column was developed over 20 min with a gradient of acetonitrile (2–80% v/v) in 0.1% v/v formic acid. The analytic column outlet was directly interfaced via an electrospray ionization source, with a hybrid quadrupole time-of-flight mass spectrometer (Xevo G2, Waters). Data were acquired over a m/z range of 300-2,000, in positive-ion mode with a cone voltage of 30 V. Scans were summed together manually and deconvoluted using MaxEnt1 (Masslynx, Waters). The theoretical molecular weights of proteins with ncAAs was calculated by first computing the theoretical molecular weight of wild-type protein using an online tool (http://web.expasy.org/protparam/) and then manually correcting for the theoretical molecular weight of ncAAs.

## Extended Data

**Figure:**
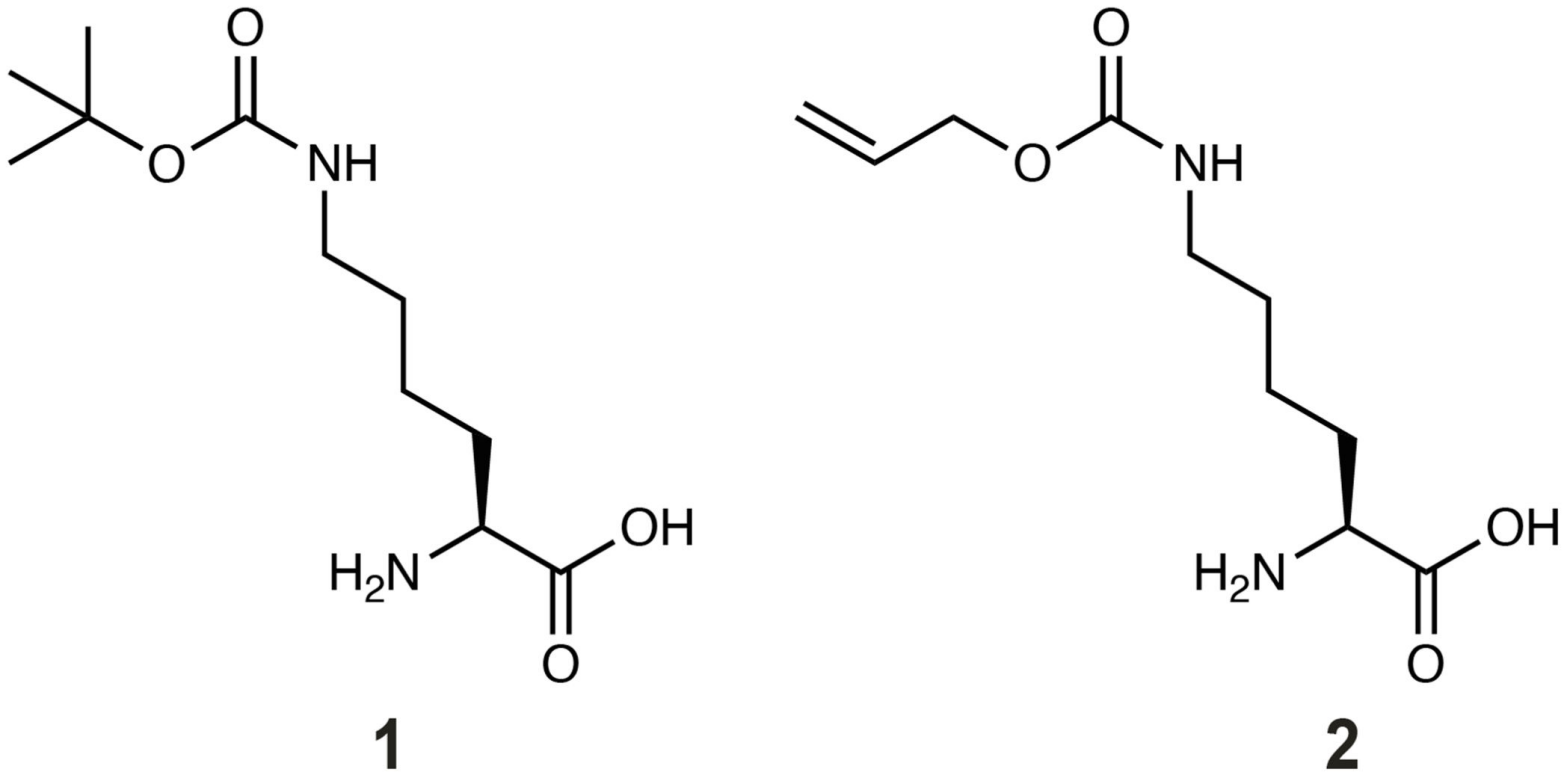


**Figure:**
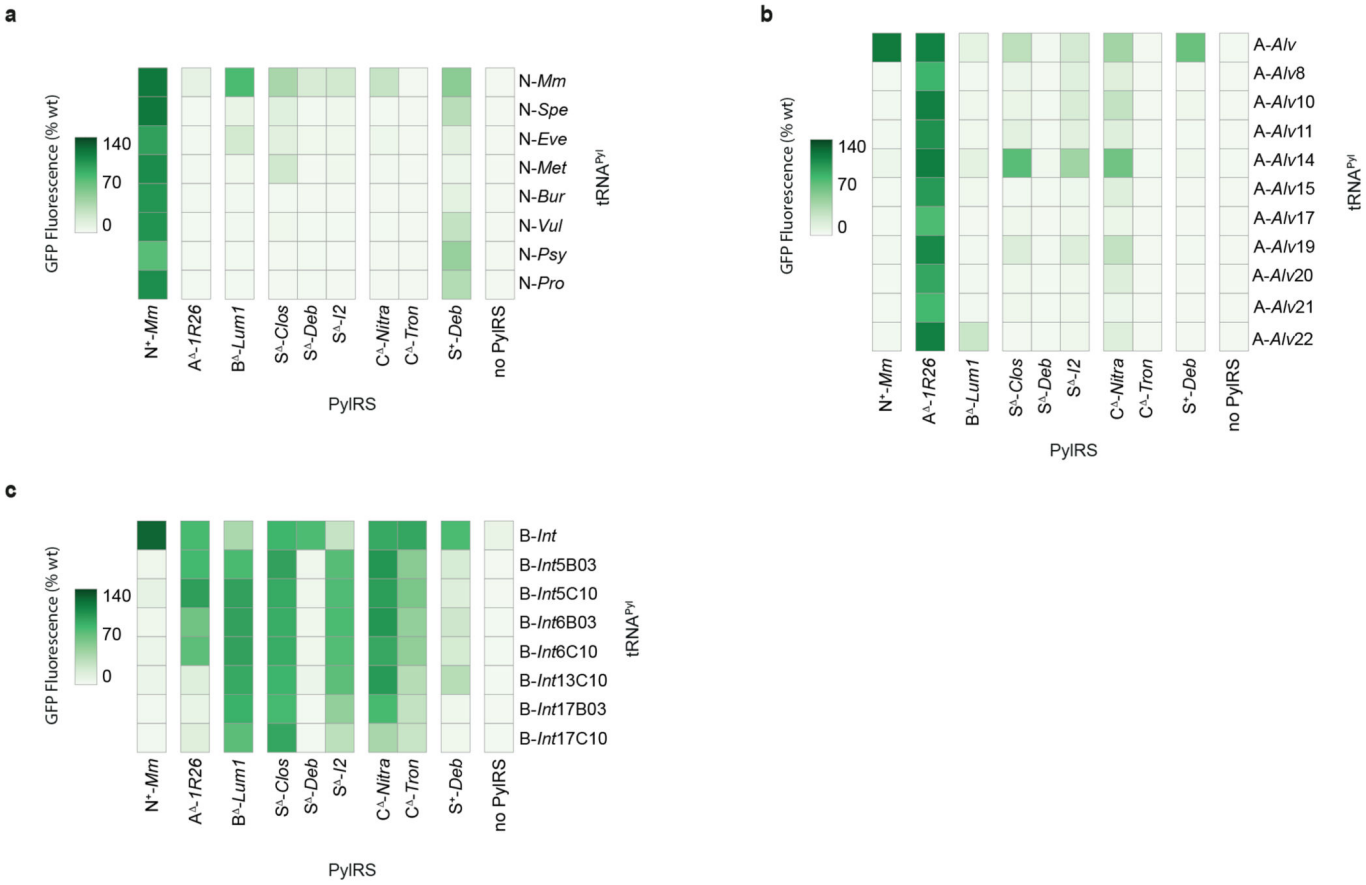


**Figure:**
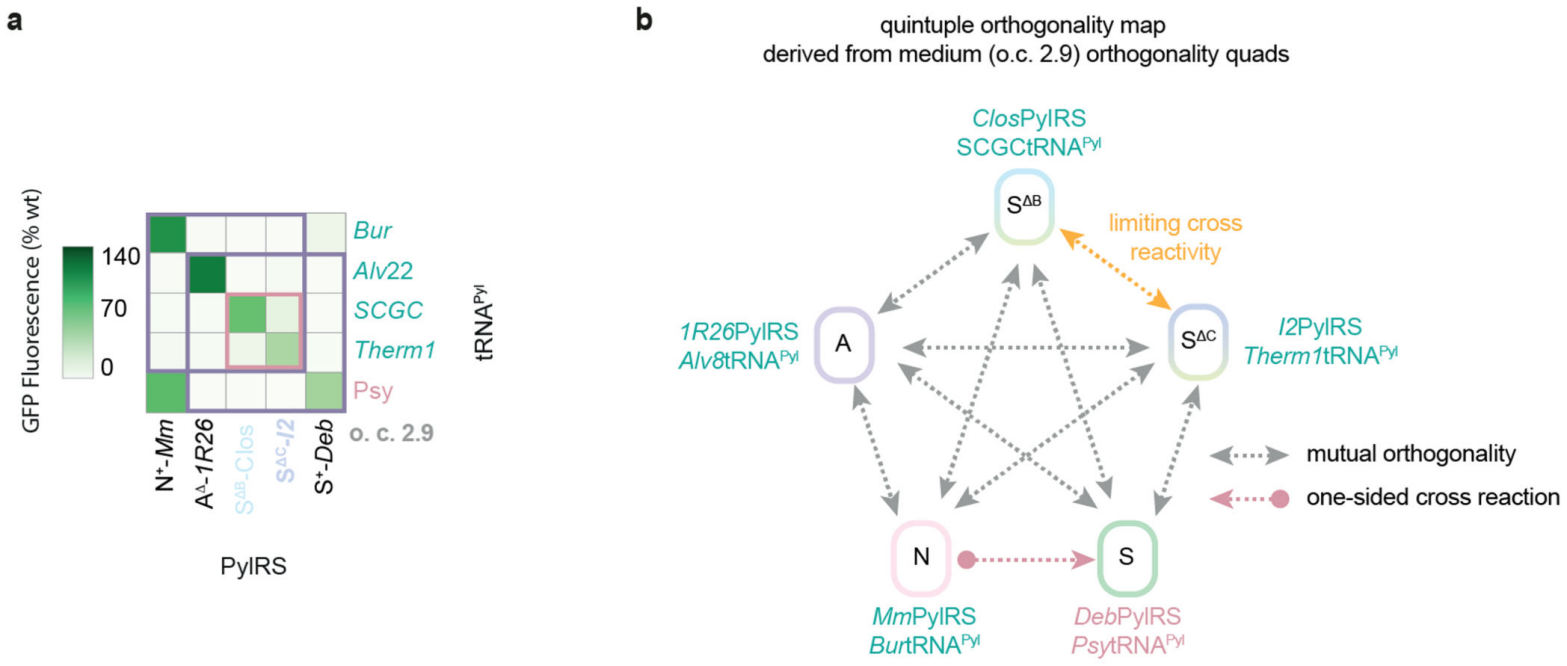


**Figure:**
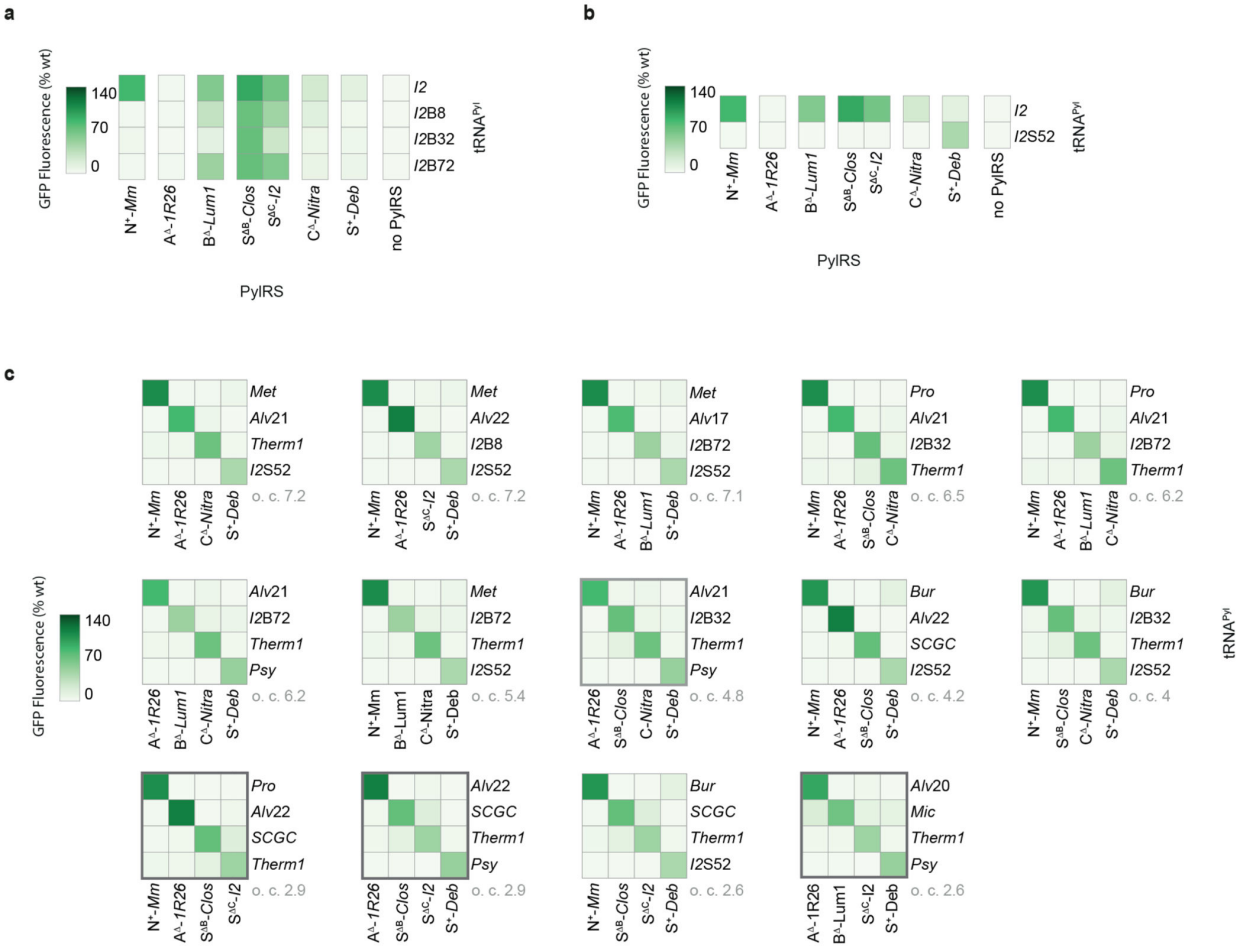


## Supplementary Material

Supplementary Notes 1-3, Figs 1-10 & Tables 4-5

Supplementary Tables 1 2 3 6 & 7

## Data Availability

All materials generated or analysed in this study are available from the corresponding author upon reasonable request. All generated data sets are provided in the supplementary information. Protein and nucleotide sequences were obtained from the NCBI Protein and NCBI Nucleotide databases, respectively.

## Code availability

The code for PylRS and tRNA^Pyl^ clustering and mutually orthogonal PylRS/tRNA^Pyl^ pair identification is available at https://github.com/JWChin-Lab/Quint-Pyl.
