# Supplementary material for: Quintuply orthogonal pyrrolysyl-tRNA synthetase/tRNAPyl pairs
Source: Nat Chem. Author manuscript; Available in PMC 2023 Nov 10. (PMC7615293; doi:10.1038/s41557-023-01232-y)
Supplement: Supplementary Notes 1-3, Figs 1-10 & Tables 4-5 [file EMS190639-supplement-Supplementary_Notes_1_3__Figs_1_10___Tables_4_5.pdf]

nature chemistry

Article https://doi.org/10.1038/s41557-023-01232-y

# Quintuply orthogonal pyrrolysyl-tRNA synthetase/tRNA$^{Pyl}$ pairs

In the format provided by the authors and unedited

**Table of Contents**


Supplementary Note 1 ........ 2

Supplementary Note 2 ........ 2

Supplementary Note 3 ........ 2

Supplementary Figure 1 ........ 4

Supplementary Figure 2 ........ 5

Supplementary Figure 3 ........ 6

Supplementary Figure 4 ........ 7

Supplementary Figure 5 ........ 9

Supplementary Figure 6 ........ 10

Supplementary Figure 7 ........ 11

Supplementary Figure 8 ........ 12

Supplementary Figure 9 ........ 13

Supplementary Figure 10 ........ 14

Supplementary Table 1 ........ 15

Supplementary Table 2 ........ 15

Supplementary Table 3 ........ 15

Supplementary Table 4 ........ 15

Supplementary Table 5 ........ 16

Supplementary Table 6 ........ 16

Supplementary Table 7 ........ 16

**Supplementary Note 1**

Aminoacyl-tRNA synthetase/tRNA pairs which have been used to incorporate non canonical amino acids (ncAAs) include *Methanococcus janaschii (Mj)* tyrosyl–tRNA synthetase (TyrRS)/*Mj*tRNA$^{\mathrm{Tyr}}$, *Archaeoglobus fulgidus (Af)* TyrRS/*Af*tRNA$^{\mathrm{Tyr}}$, *Methanococcus maripaludis* (*Mmp*) phosphoseryl–tRNA synthetase (SepRS)/*Mj*tRNA$^{\mathrm{Sep}}$, *Saccharomyces cerevisiae* (*Sc*) tryptophanyl–tRNA synthetase (TrpRS)/*Sc*tRNA$^{\mathrm{Trp}}$), *Methanosarcina mazei* (*Mm*) or *Methanosarcina barkeri* (*Mb*) pyrrolysyl–tRNA synthetase (PylRS)/*Mm*tRNA$^{\mathrm{Pyl}}$ or *Mb*tRNA$^{\mathrm{Pyl}}$, and engineered mutually orthogonal PylRS/tRNA$^{\mathrm{Pyl}}$ pairs such as *Candidatus Methanomethylophilus sp.1R26* (*1R26*)PylRS/*Candidatus Methanomethylophilus alvus* (*Alv*)tRNA$^{\mathrm{Pyl\text{-}8}}$ and *Methanomassiliicoccus luminyensis 1* (*Lum1*)PylRS/*Candidatus Methanomassiliicoccus intestinalis* (*Int*)tRNA$^{\mathrm{Pyl\text{-}17C10}}$.[1-12]

**Supplementary Note 2**

Certain class S pyl tRNAs contain 6-8 base pair D loops, while several class C pyl tRNAs contain long variable loops. Previous studies have shown that structural elements can strongly influence tRNA$^{\mathrm{Pyl}}$:PylRS interactions;[3,13] indeed, the orthogonality of the PylRS/tRNA$^{\mathrm{Pyl}}$ system with respect to endogenous aminoacyl-tRNA synthetases in a variety of host organisms has been attributed to the compact structure of the tRNA$^{\mathrm{Pyl}}$ body.[14] Notably, expansions of the tRNA$^{\mathrm{Pyl}}$ variable loop have previously been used to attenuate cross-reactivity by a non-cognate PylRS class, while maintaining activity with the cognate PylRS class.[2,3,15,16] Moreover, multiple pyl tRNAs from both classes contain unusual (adenine or uracil) nucleobases at the discriminator base position, which is a known identity element for previously characterised PylRS proteins.[17,18]

**Supplementary Note 3**

Six PylRS enzymes (C$^{\Delta}$-*Nitrososphaeria archaeon* (*Nitra*)PylRS, C$^{\Delta}$-*Methanonatronarchaeia archaeon* (*Tron*)PylRS, S$^{\Delta}$-*Desulfosporosinus sp. I2* (*I2*)PylRS, S$^{\Delta}$-*Clostridiales bacterium* (*Clos*)PylRS, S$^{\Delta}$-*Deltaproteobacteria bacterium* (*Deb*)PylRS, and S$^{\Delta}$-*Spirochaetales bacterium* (*Spi*)PylRS) led to GFP production, at a level at least 50% of the wtGFP control, in the presence of the appropriate class C or S tRNA$^{\mathrm{Pyl}}$.

Intriguingly, the active class C PylRS enzymes showed considerable specificity towards certain class C pyl tRNAs over class S pyl tRNAs. In particular, C-*Tron*PylRS, which was highly active with the C-*Tron*tRNA$^{\mathrm{Pyl}}$ (76% of wtGFP control), had less than 10% activity with all but one class S tRNA$^{\mathrm{Pyl}}$ tested.

Most active class S PylRS enzymes aminoacylated pyl tRNAs within both class C and class S. However, C-*Candidatus Methanohalarchaeum thermophilum 1* (*Therm1*)$tRNA^{Pyl}$ (and to a lesser extent, C-*Candidate division MSBL1 archaeon SCGC-AAA382A20* (*SCGC*)$tRNA^{Pyl}$) was poorly recognised by most active class S PylRS enzymes but formed a highly active pair with $C^{\Delta}$-*Nitra*PylRS.

With regard to the previously characterised pyl tRNAs,[2,3] most class C and class S PylRS enzymes proved highly active with B-*Int*$tRNA^{Pyl}$, giving rise to GFP levels over 80% of the wtGFP control in some cases. In addition, several class C and class S PylRS enzymes also showed moderate to strong activity with N-*Mm*$tRNA^{Pyl}$ and $A^{\Delta}$-*Alv*$tRNA^{Pyl}$. Of the previously characterised PylRS enzymes, $N^{+}$-*Mm*PylRS was by far the most promiscuous, giving rise to over 50% of wild-type GFP production levels in the presence of eleven out of 16 pyl tRNAs (including all but two class S pyl tRNAs). These included S-*Clos*$tRNA^{Pyl}$ and S-*Deb*$tRNA^{Pyl}$, which showed only modest activity with the most active class S PylRS enzymes. To a lesser extent, class A and class B PylRS enzymes also cross-reacted with certain class S and class C pyl tRNAs. Despite this, we were pleased to observe that C-*SCGC*$tRNA^{Pyl}$ and C-*Therm1*$tRNA^{Pyl}$ were orthogonal to $N^{+}$-*Mm*PylRS, $A^{\Delta}$-*1R26*PylRS, and $B^{\Delta}$-*Lum1*PylRS; this demonstrated that naturally occurring $tRNA^{Pyl}$ can be found that are orthogonal to PylRS enzymes taken from all other classes.

Two wild-type class S PylRS enzymes, $S^{+}$- *Gemmatimonadetes bacterium* (*Gem*)PylRS and $S^{+}$-*Deb*PylRS, were expressed and showed convincing activity. $S^{+}$-*Gem*PylRS exhibited similar $tRNA^{Pyl}$ specificity to its $S^{\Delta}$ variant. However, $S^{+}$-*Deb*PylRS, the most active $S^{+}$ system characterised, showed a markedly different activity profile to $S^{\Delta}$-*Deb*PylRS, for instance having much higher activity with A-*Alv*$tRNA^{Pyl}$ (72% versus 2% of wtGFP control, respectively), but much lower activity with C-*Tron*$tRNA^{Pyl}$ (10% vs 64%). This is consistent with reports that PylSn proteins modulate $tRNA^{Pyl}$ specificity.[19,20]

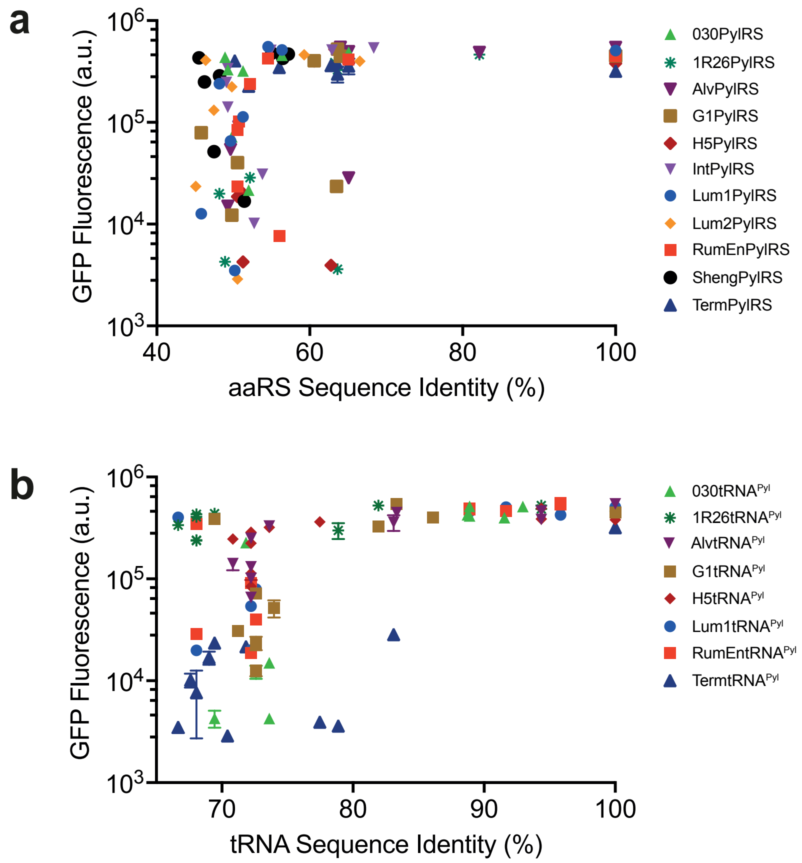


**Supplementary Figure 1**

**a.** Activity of each combination of ΔN PylRS$_i$ (legend) and ΔN tRNA$^{Pyl}{}_j$, measured by production of GFP(150AllocK)$_{His6}$ from cells bearing a *GFP(150TAG)$_{His6}$* gene in the presence of 4 mM AllocK **1**, plotted against the sequence identity between ΔN PylRS$_i$ and ΔN PylRS$_j$, where ΔN PylRS$_j$ is the synthetase from the same organism as ΔN tRNA$^{Pyl}{}_j$. **b.** Activity of each combination ΔN PylRS$_i$ and ΔN tRNA$^{Pyl}{}_j$ (legend), plotted against the sequence identity between ΔN tRNA$^{Pyl}{}_i$ and ΔN tRNA$^{Pyl}{}_j$, where ΔN tRNA$^{Pyl}{}_i$ is the tRNA$^{Pyl}$ from the same organism as ΔN PylRS$_i$. Dots represent the mean of three biological replicates, error bars show ± s.d.. All numerical values are provided (**Supplementary Table 2**).

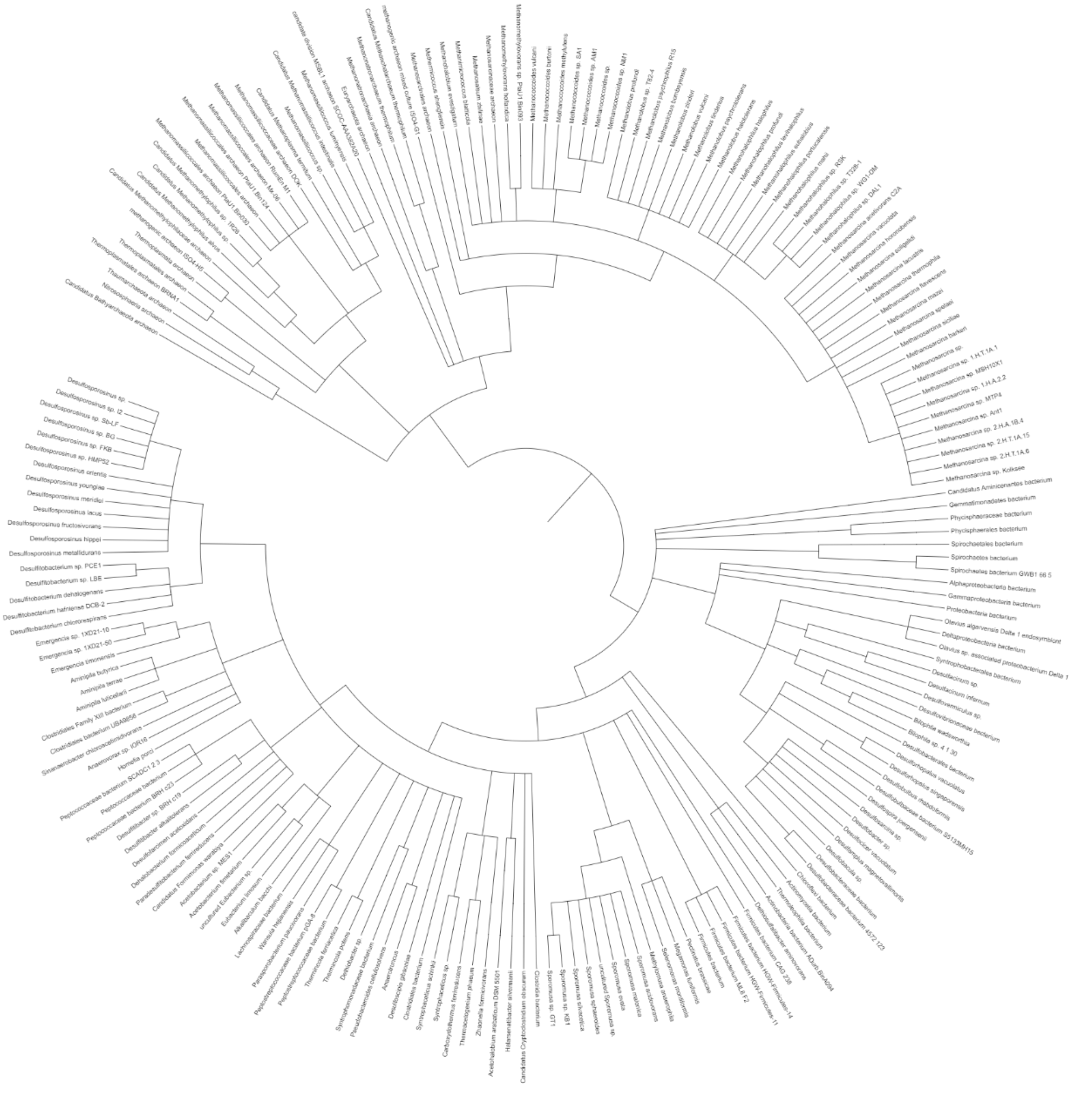

**Supplementary Figure 2**

Schematic representation of the phylogeny of the organisms containing the 351 pyrrolysine systems discovered, generated with iTOL *v6* software based on the unique taxonomic identifiers of the PylRS sequences. The full phylogenetic tree is provided as .txt file and can be interactively studied using *iTOL v6* software or equivalent programs.

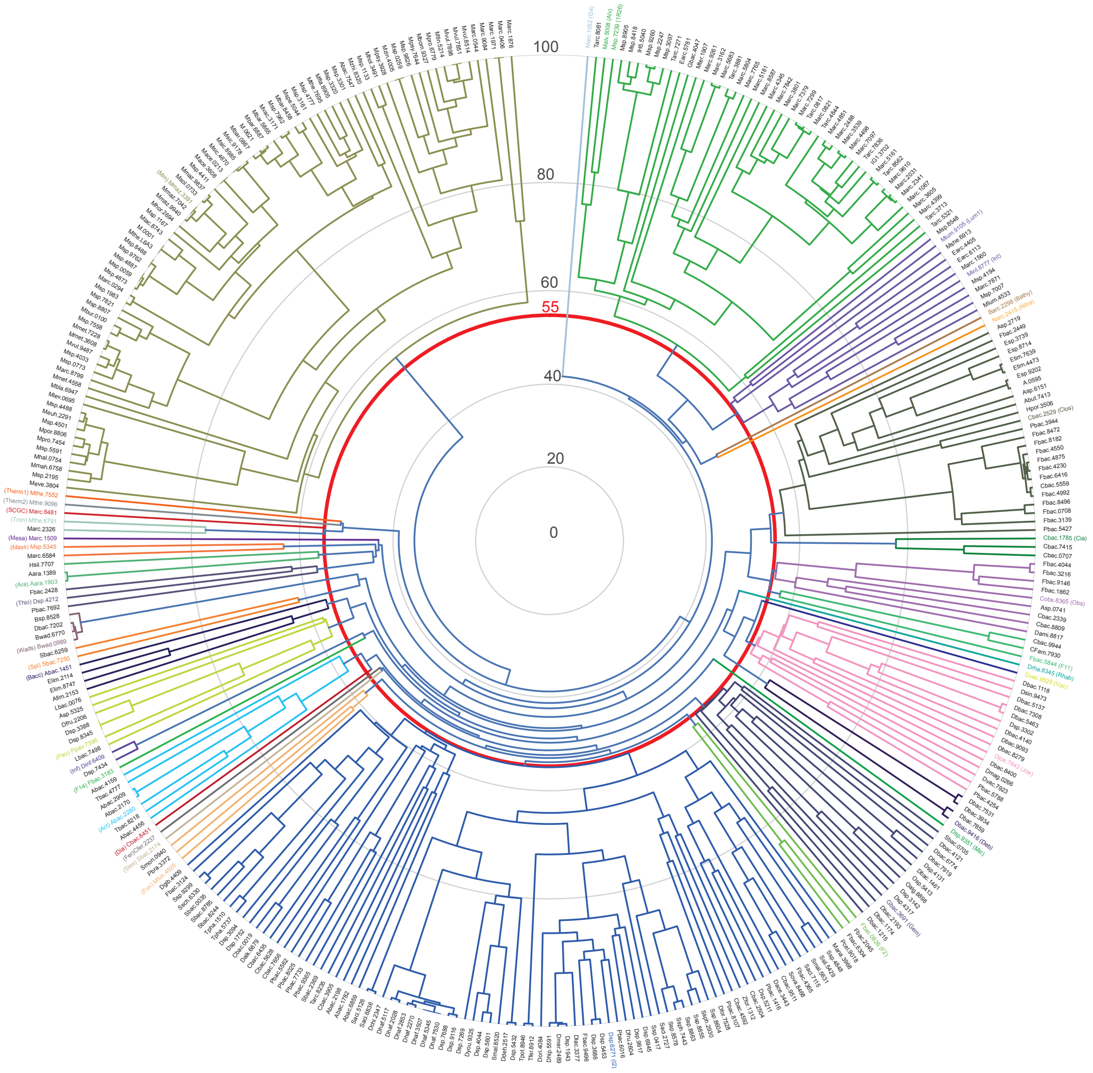


**Supplementary Figure 3**

Fully annotated dendrogram showing the 37 clusters generated from agglomerative hierarchical clustering of the 351 PylRS C-terminal domain amino acid sequences, labelled with the identifiers indexed in **Supplementary Table 1**. Coloured labels correspond to the PylRS sequences chosen as cluster representatives. The radial coordinate represents percentage sequence identity (log scale), with grey contours corresponding to intervals of 20%. The red contour represents 55% sequence identity, the clustering threshold value. Unweighted average linkage clustering was performed using the *scikit-learn* package (version 1.0.1) in the *Python* programming language (version 3.9.7), with sequence identity scores converted to Euclidian distance measures.

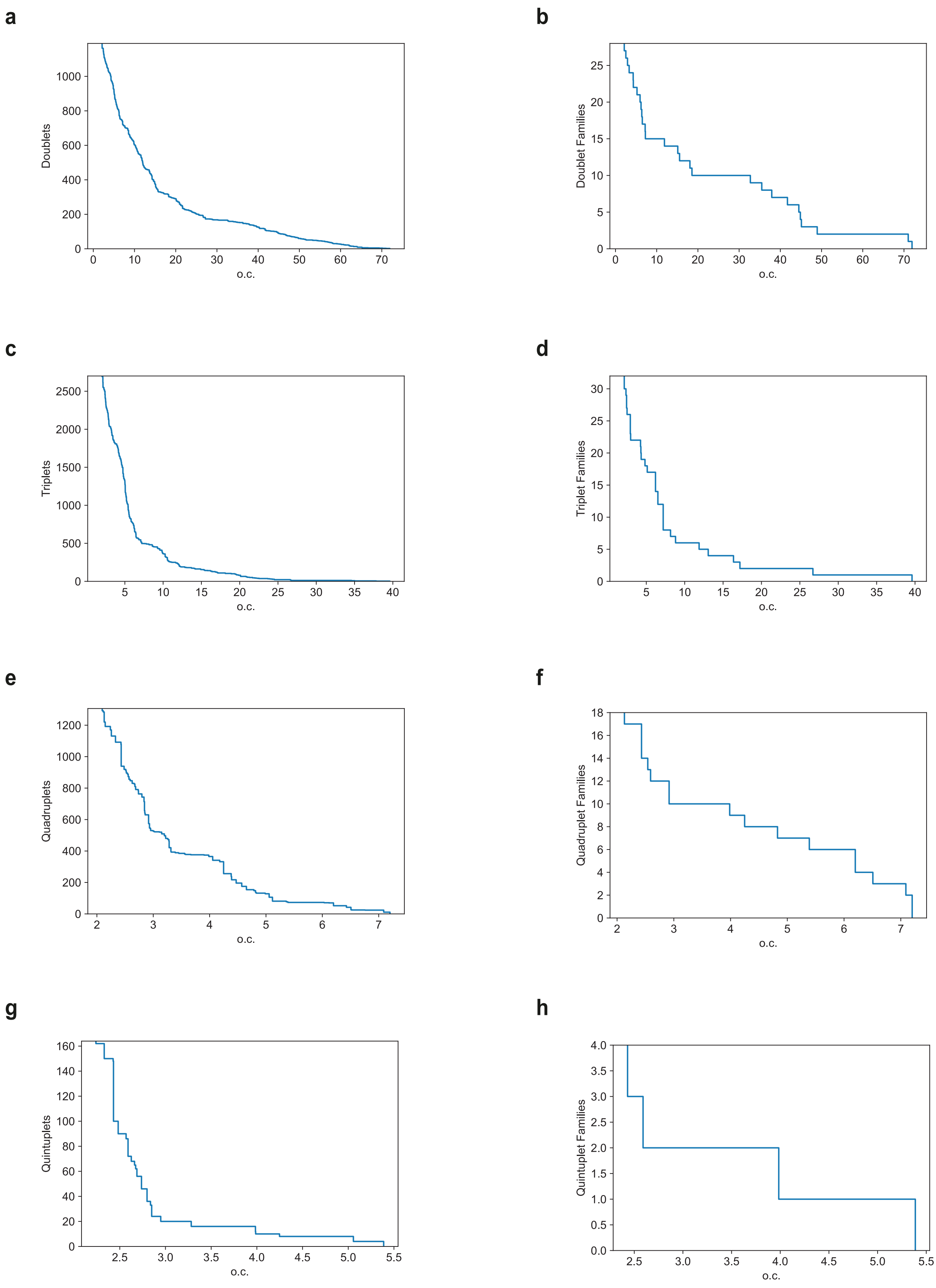


**Supplementary Figure 4**

**a.** Plot of the number of mutually orthogonal PylRS/tRNA$^{Pyl}$ doublets against orthogonality coefficient (o.c.). **b.** Plot of the number of mutually orthogonal PylRS/tRNA$^{Pyl}$ doublet families against orthogonality coefficient. **c.** Plot of the number of mutually orthogonal PylRS/tRNA$^{Pyl}$ triplets against

orthogonality coefficient. **d.** Plot of the number of mutually orthogonal PylRS/tRNA$^{Pyl}$ triplet families against orthogonality coefficient. **e.** Plot of the number of mutually orthogonal PylRS/tRNA$^{Pyl}$ quadruplets against orthogonality coefficient. **f.** Plot of the number of mutually orthogonal PylRS/tRNA$^{Pyl}$ quadruplet families against orthogonality coefficient. **g.** Plot of the number of mutually orthogonal PylRS/tRNA$^{Pyl}$ quintuplets against orthogonality coefficient. **h.** Plot of the number of mutually orthogonal PylRS/tRNA$^{Pyl}$ quintuplet families against orthogonality coefficient. Orthogonality coefficient (o.c.) is the quotient of the lowest intra-pair activity over the highest inter-pair cross-reactivity. Sets of pairs were considered mutually orthogonal if the lowest intra-pair activity was greater than 40% of the wtGFP control, the highest inter-pair cross-reactivity was less than 20% of the wtGFP control, and the o.c. was higher than 2.5. We grouped mutually orthogonal sets together into families if they involved the same PylRS enzymes.

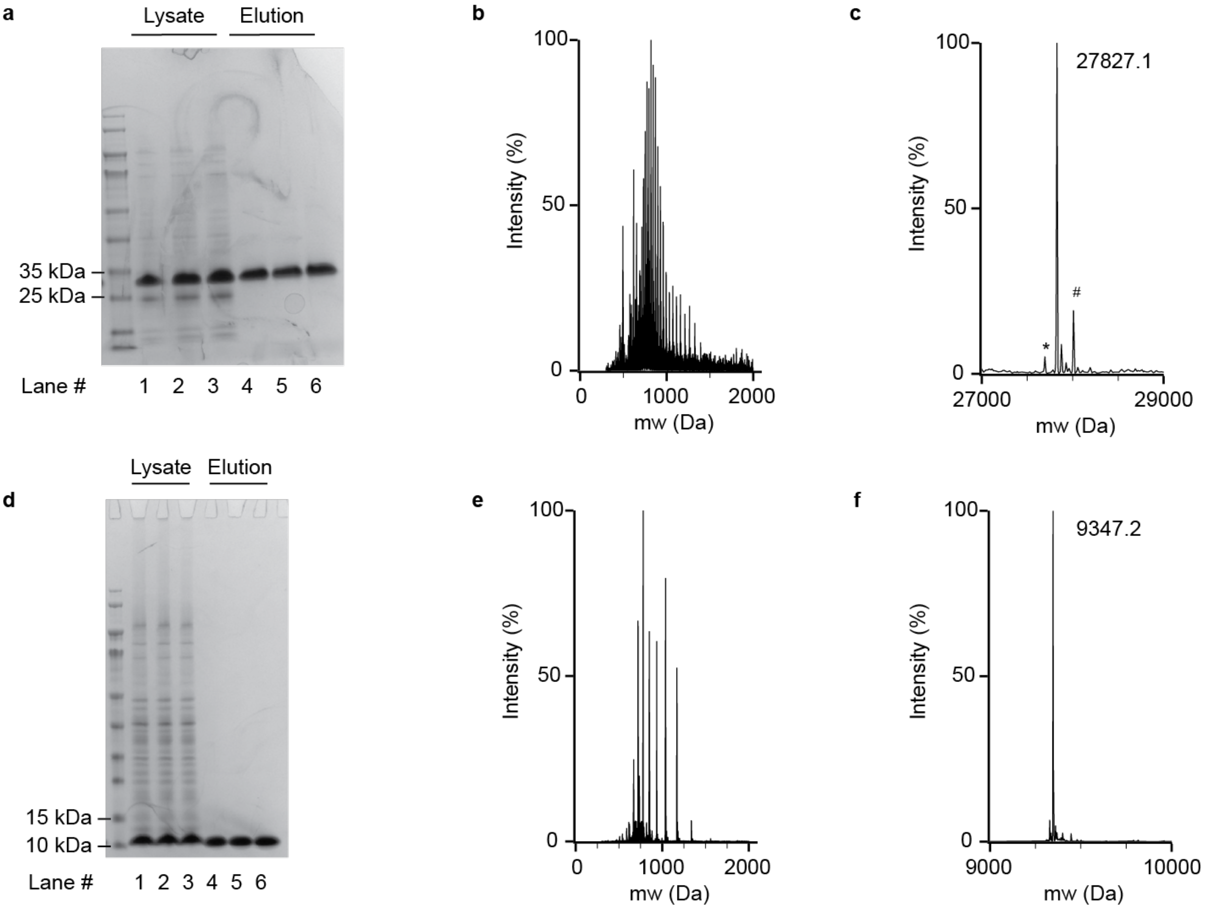


**Supplementary Figure 5**

**a.** Coomassie stained SDS PAGE gel of cell lysate (lanes 1-3) and Nickel NTA purified GFP$_{His6}$ (lanes 4-6) from DH10B *E.coli* cells expressing *GFP$_{His6}$* in the presence of *Mm*PylRS/*Alv*tRNA$^{Pyl-21}$ and 4 mM AllocK **1**. **b.** Raw mass spectrum of the purified GFP$_{His6}$ shown in panel **a**. The experiment was carried out in biological triplicates with similar results. Peak lists for all replicates are given in **Supplementary Table 6**. **c.** Deconvoluted mass spectrum of the purified GFP$_{His6}$ shown in panel **a**. Expected mass after dehydration 27827.3, mass found 27827.1. Peak labeled with an asterisk corresponds to loss of methionine. Peak labeled with a hash corresponds to the 4-(2-Aminoethyl) benzenesulfonyl adduct (+183 m/z) resulting from the incubation with *cOmplete* proteinase inhibitor during purification. **d.** Coomassie stained SDS PAGE gel of cell lysate (lanes 1-3) and Nickel NTA purified Ub(11Ser)$_{His6}$ (lanes 4-6) from DH10B *E.coli* cells expressing *Ub(11TCA)$_{His6}$* in the presence of *Mm*PylRS/*Alv*tRNA$^{Pyl-21}$ and 4 mM AllocK **1**. **e.** Raw mass spectrum of the purified Ub(11Ser)$_{His6}$ shown in panel **d**. The experiment was carried out in biological triplicates with similar results. Peak lists for all replicates are given in **Supplementary Table 6**. **f.** Deconvoluted mass spectrum of the purified Ub(11Ser)$_{His6}$ shown in panel **d**. Expected mass 9346.6, mass found 9347.2.

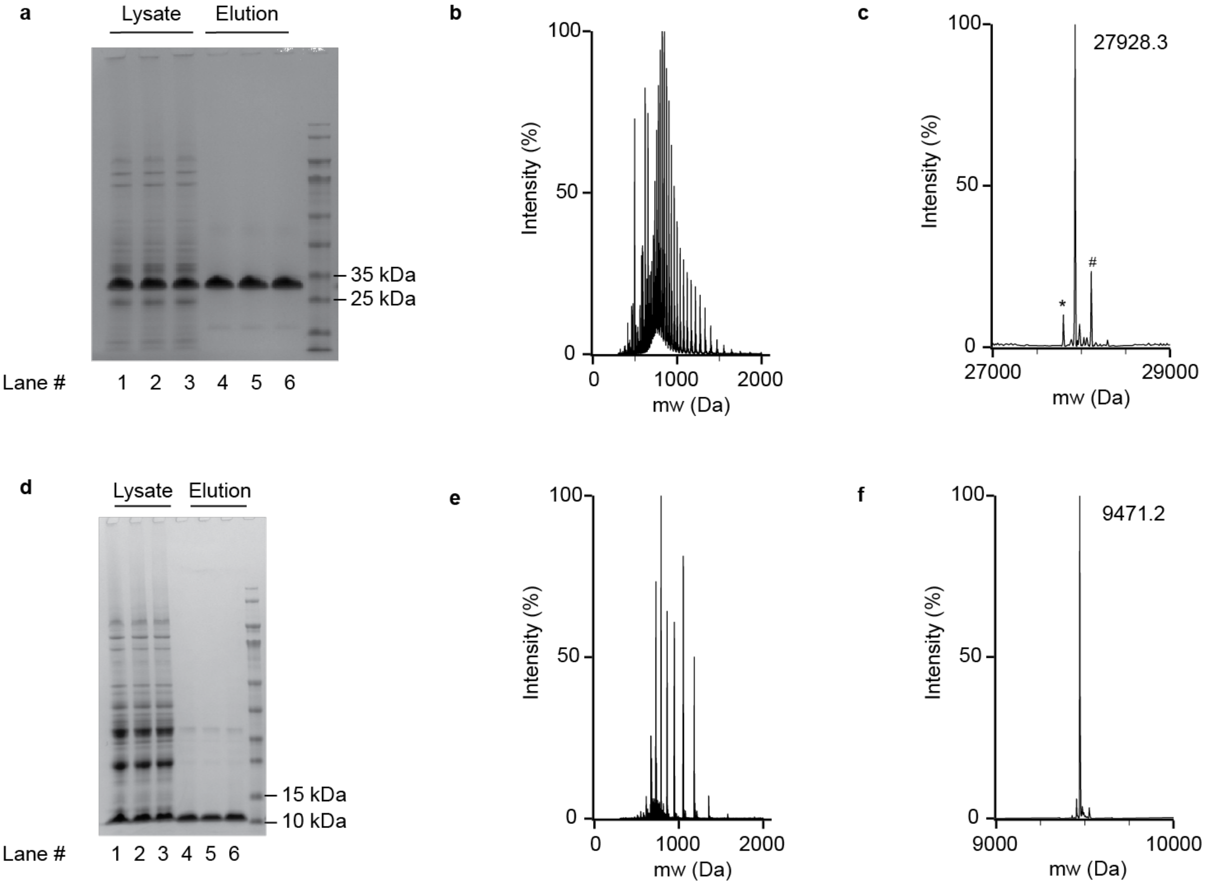


**Supplementary Figure 6**

**a.** Coomassie stained SDS PAGE gel of cell lysate (lanes 1-3) and Nickel NTA purified GFP(150AllocK)$_{His6}$ (lanes 4-6) from DH10B *E.coli* cells expressing *GFP(150TAG)$_{His6}$* in the presence of *1R26*PylRS/*Alv*tRNA$^{Pyl-21}$ and 4 mM AllocK **1**. **b.** Raw mass spectrum of the purified GFP(150AllocK)$_{His6}$ shown in panel **a**. The experiment was carried out in biological triplicates with similar results. Peak lists for all replicates are given in **Supplementary Table 6**. **c.** Deconvoluted mass spectrum of the purified GFP(150AllocK)$_{His6}$ shown in panel **a**. Expected mass after dehydration 27925.4, mass found 27928.3. Peak labeled with an asterisk corresponds to loss of methionine. Peak labeled with a hash corresponds to the 4-(2-Aminoethyl) benzenesulfonyl adduct (+183 m/z) resulting from the incubation with *cOmplete* proteinase inhibitor during purification. **d.** Coomassie stained SDS PAGE gel of cell lysate (lanes 1-3) and Nickel NTA purified Ub(11AllocK)$_{His6}$ (lanes 4-6) from DH10B *E.coli* cells expressing *Ub(11TAG)$_{His6}$* in the presence of *1R26*PylRS/*Alv*tRNA$^{Pyl-21}$ and 4 mM AllocK **1**. **e.** Raw mass spectrum of the purified Ub(11AllocK)$_{His6}$ shown in panel **d**. The experiment was carried out in biological triplicates with similar results. Peak lists for all replicates are given in **Supplementary Table 6**. **f.** Deconvoluted mass spectrum of the purified Ub(11AllocK)$_{His6}$ shown in panel **d**. Expected mass 9471.7, mass found 9471.2.

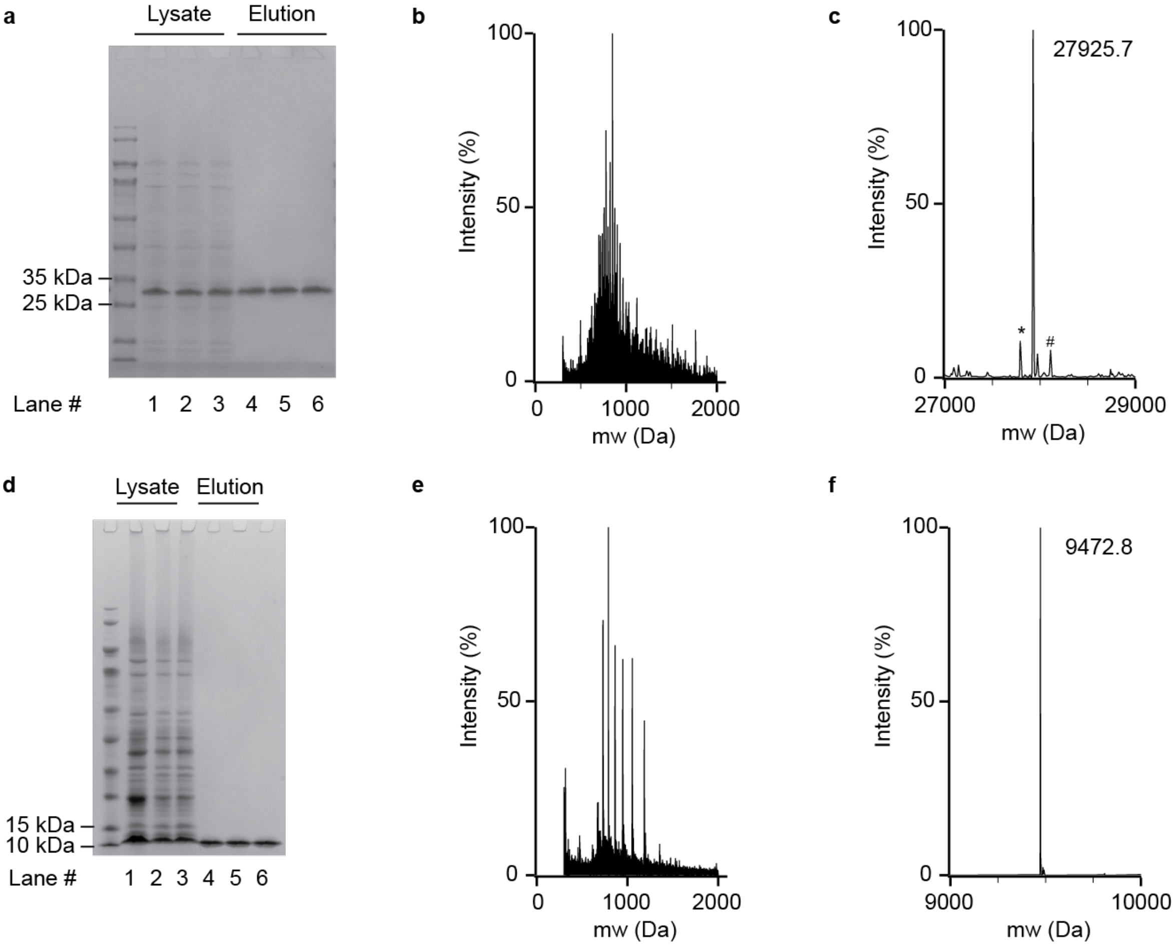


**Supplementary Figure 7**

**a.** Coomassie stained SDS PAGE gel of cell lysate (lanes 1-3) and Nickel NTA purified GFP(150AllocK)$_{His6}$ (lanes 4-6) from DH10B *E.coli* cells expressing *GFP(150TAG)*$_{His6}$ in the presence of *Deb*PylRS/*I2*tRNA$^{Pyl-S52}$ and 4 mM AllocK **1**. **b.** Raw mass spectrum of the purified GFP(150AllocK)$_{His6}$ shown in panel **a**. The experiment was carried out in biological triplicates with similar results. Peak lists for all replicates are given in **Supplementary Table 6**. **c.** Deconvoluted mass spectrum of the purified GFP(150AllocK)$_{His6}$ shown in panel **a**. Expected mass after dehydration 27925.4, mass found 27925.7. Peak labeled with an asterisk corresponds to loss of methionine. Peak labeled with a hash corresponds to the 4-(2-Aminoethyl) benzenesulfonyl adduct (+183 m/z) resulting from the incubation with *cOmplete* proteinase inhibitor during purification. **d.** Coomassie stained SDS PAGE gel of cell lysate (lanes 1-3) and Nickel NTA purified Ub(11AllocK)$_{His6}$ (lanes 4-6) from DH10B *E.coli* cells expressing *Ub(11TAG)*$_{His6}$ in the presence of *Deb*PylRS/*I2*tRNA$^{Pyl-S52}$ and 4 mM AllocK **1**. **e.** Raw mass spectrum of the purified Ub(11AllocK)$_{His6}$ shown in panel **d**. The experiment was carried out in biological triplicates with similar results. Peak lists for all replicates are given in **Supplementary Table 6**. **f.** Deconvoluted mass spectrum of the purified Ub(11AllocK)$_{His6}$ shown in panel **d**. Expected mass 9471.7, mass found 9472.8.

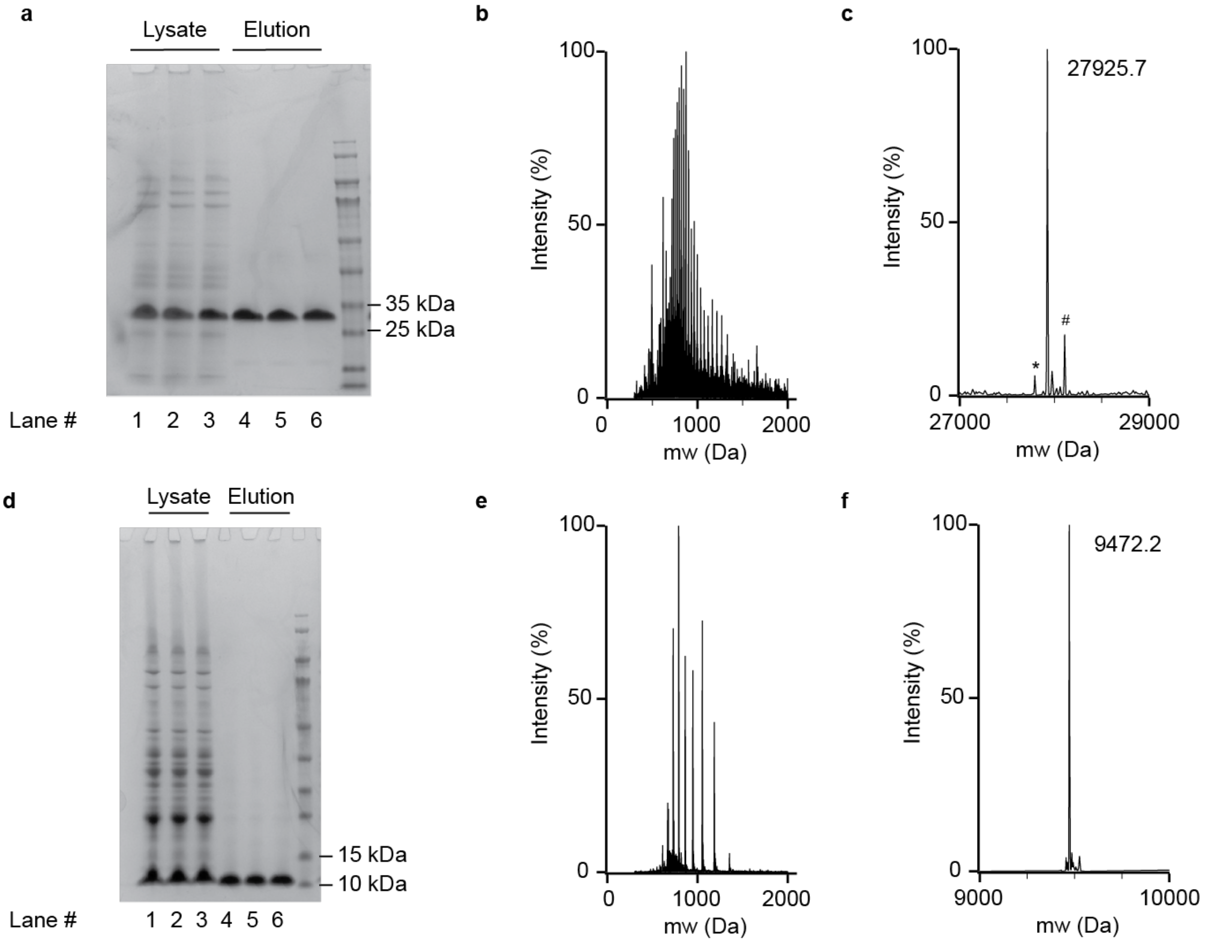


**Supplementary Figure 8**

**a.** Coomassie stained SDS PAGE gel of cell lysate (lanes 1-3) and Nickel NTA purified GFP(150AllocK)$_{\text{His6}}$ (lanes 4-6) from DH10B *E.coli* cells expressing *GFP(150TAG)*$_{His6}$ in the presence of *Lum1*PylRS/*I2*tRNA$^{\text{Pyl-B72}}$ and 4 mM AllocK **1**. **b.** Raw mass spectrum of the purified GFP(150AllocK)$_{\text{His6}}$ shown in panel **a**. The experiment was carried out in biological triplicates with similar results. Peak lists for all replicates are given in **Supplementary Table 6**. **c.** Deconvoluted mass spectrum of the purified GFP(150AllocK)$_{\text{His6}}$ shown in panel **a**. Expected mass after dehydration 27925.4, mass found 27925.7. Peak labeled with an asterisk corresponds to loss of methionine. Peak labeled with a hash corresponds to the 4-(2-Aminoethyl) benzenesulfonyl adduct (+183 m/z) resulting from the incubation with *cOmplete* proteinase inhibitor during purification. **d.** Coomassie stained SDS PAGE gel of cell lysate (lanes 1-3) and Nickel NTA purified Ub(11AllocK)$_{\text{His6}}$ (lanes 4-6) from DH10B *E.coli* cells expressing *Ub(11TAG)*$_{His6}$ in the presence of *Lum1*PylRS/*I2*tRNA$^{\text{Pyl-B72}}$ and 4 mM AllocK **1**. **e.** Raw mass spectrum of the purified Ub(11AllocK)$_{\text{His6}}$ shown in panel **d**. The experiment was carried out in biological triplicates with similar results. Peak lists for all replicates are given in **Supplementary Table 6**. **f.** Deconvoluted mass spectrum of the purified Ub(11AllocK)$_{\text{His6}}$ shown in panel **d**. Expected mass 9471.7, mass found 9472.2.

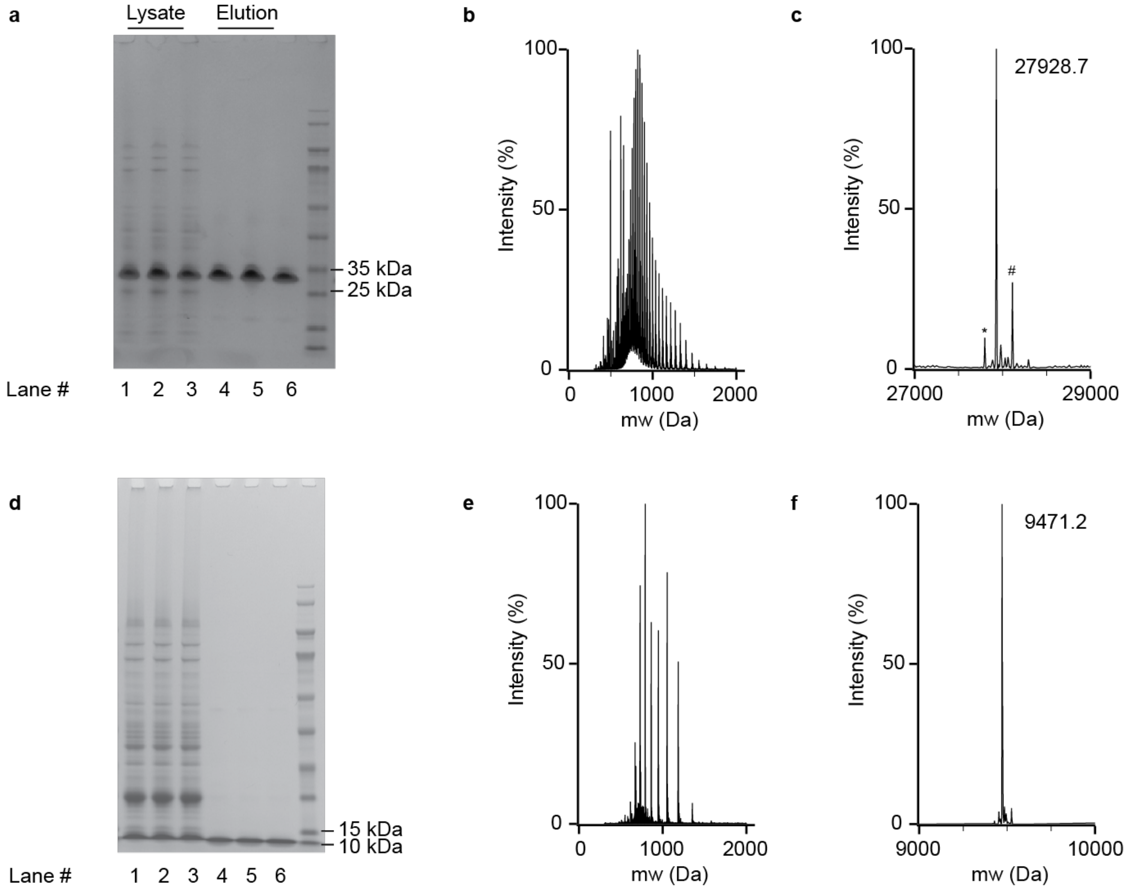


**Supplementary Figure 9**

**a.** Coomassie stained SDS PAGE gel of cell lysate (lanes 1-3) and Nickel NTA purified GFP(150AllocK)$_{His6}$ (lanes 4-6) from DH10B *E.coli* cells expressing *GFP(150TAG)$_{His6}$* in the presence of *Mm*PylRS/*Met*tRNA$^{Pyl}$ and 4 mM AllocK **1**. **b.** Raw mass spectrum of the purified GFP(150AllocK)$_{His6}$ shown in panel **a**. The experiment was carried out in biological triplicates with similar results. Peak lists for all replicates are given in **Supplementary Table 6**. **c.** Deconvoluted mass spectrum of the purified GFP(150AllocK)$_{His6}$ shown in panel **a**. Expected mass after dehydration 27925.4, mass found 27928.7. Peak labeled with an asterisk corresponds to loss of methionine. Peak labeled with a hash corresponds to the 4-(2-Aminoethyl) benzenesulfonyl adduct (+183 m/z) resulting from the incubation with *cOmplete* proteinase inhibitor during purification. **d.** Coomassie stained SDS PAGE gel of cell lysate (lanes 1-3) and Nickel NTA purified Ub(11AllocK)$_{His6}$ (lanes 4-6) from DH10B *E.coli* cells expressing *Ub(11TAG)$_{His6}$* in the presence of *Mm*PylRS/*Met*tRNA$^{Pyl}$ and 4 mM AllocK **1**. **e.** Raw mass spectrum of the purified Ub(11AllocK)$_{His6}$ shown in panel **d**. The experiment was carried out in biological triplicates with similar results. Peak lists for all replicates are given in **Supplementary Table 6**. **f.** Deconvoluted mass spectrum of the purified Ub(11AllocK)$_{His6}$ shown in panel **d**. Expected mass 9471.7, mass found 9471.2.

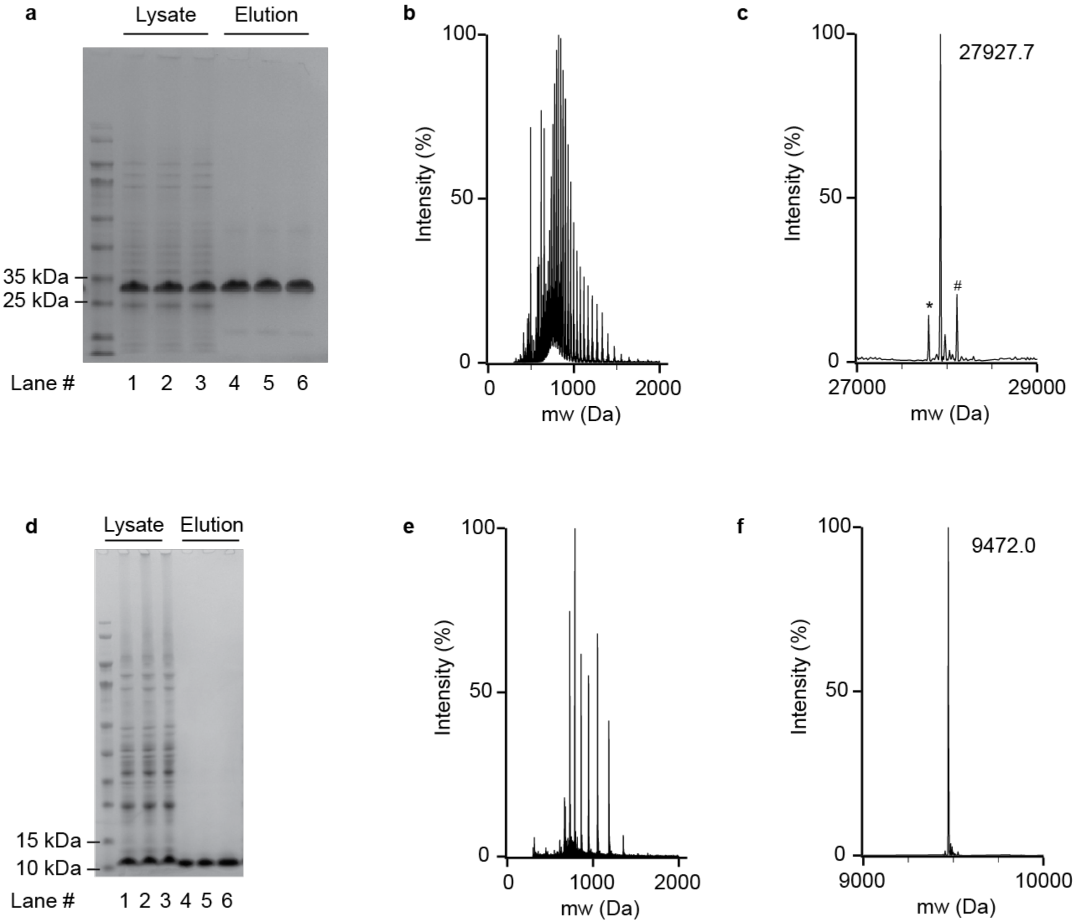


**Supplementary Figure 10**

**a.** Coomassie stained SDS PAGE gel of cell lysate (lanes 1-3) and Nickel NTA purified GFP(150AllocK)$_{His6}$ (lanes 4-6) from DH10B *E.coli* cells expressing *GFP(150TAG)$_{His6}$* in the presence of *Nitra*PylRS/*Therm1*tRNA$^{Pyl}$ and 4 mM AllocK **1**. **b.** Raw mass spectrum of the purified GFP(150AllocK)$_{His6}$ shown in panel **a**. The experiment was carried out in biological triplicates with similar results. Peak lists for all replicates are given in **Supplementary Table 6**. **c.** Deconvoluted mass spectrum of the purified GFP(150AllocK)$_{His6}$ shown in panel **a**. Expected mass after dehydration 27925.4, mass found 27927.7. Peak labeled with an asterisk corresponds to loss of methionine. Peak labeled with a hash corresponds to the 4-(2-Aminoethyl) benzenesulfonyl adduct (+183 m/z) resulting from the incubation with *cOmplete* proteinase inhibitor during purification. **d.** Coomassie stained SDS PAGE gel of cell lysate (lanes 1-3) and Nickel NTA purified Ub(11AllocK)$_{His6}$ (lanes 4-6) from DH10B *E.coli* cells expressing *Ub(11TAG)$_{His6}$* in the presence of *Nitra*PylRS/*Therm1*tRNA$^{Pyl}$ and 4 mM AllocK **1**. **e.** Raw mass spectrum of the purified Ub(11AllocK)$_{His6}$ shown in panel **d**. The experiment was carried out in biological triplicates with similar results. Peak lists for all replicates are given in **Supplementary Table 6**. **f.** Deconvoluted mass spectrum of the purified Ub(11AllocK)$_{His6}$ shown in panel **d**. Expected mass 9471.7, mass found 9472.0.

**Supplementary Table 1**

Database of PylRS and $tRNA^{Pyl}$ sequences together with classification resulting from hierarchical clustering. The table is provided in a separate excel sheet.

**Supplementary Table 2**

Table of numerical values of all fluorescence measurements conducted in this work. The table is provided as a separate excel sheet.

**Supplementary Table 3**

Sequence information of all DNA constructs used in this work. The table is provided as a separate excel sheet.

**Supplementary Table 4**

Isolated protein yields for the production of $GFP(150AllocK)_{His6}$ from $\mathit{GFP(150TAG)}_{His6}$ with all the PylRS/$tRNA^{Pyl}$ pairs forming the most orthogonal quintuply orthogonal set. Primary data is given in **Supplementary Table 7**.

| **Protein** | **PylRS** | **$tRNA^{Pyl}$** | **yield (mg/L)** | **% GFP(wt)** |
|---|---|---|---|---|
| $GFP(150AllocK)_{His6}$ produced from G*FP(150TAG)*$_{His6}$ | $N^{+}$-*Mm* | *Met* | 80.2 ± 13.6 | 97 |
| | $A^{\Delta}$-*1R26* | *Alv*-21 | 116.8 ± 11.6 | 141 |
| | $C^{\Delta}$-*Nitra* | *Therm1* | 102.0 ± 15.5 | 123 |
| | $S^{+}$-*Deb* | *I2*-S52 | 27.3 ± 1.6 | 33 |
| | $B^{\Delta}$-*Lum1* | *I2*-B72 | 96.1 ± 8.9 | 116 |
| $GFP_{His6}$ produced from *GFP*$_{His6}$ | $N^{+}$-*Mm* | *Alv*-21 | 82.6 ± 1.9 | 100 |

**Supplementary Table 5**

Summary of isolated protein yields for the production of Ub(11AllocK)$_{His6}$ from *Ub(11TAG)$_{His6}$* with the all PylRS/tRNA$^{Pyl}$ pairs forming the most orthogonal quintuply orthogonal set. Primary data is given in **Supplementary Table 7**.

| **Protein** | **PylRS** | **tRNA$^{Pyl}$** | **yield (mg/L)** | **% Ub(K11S)** |
|---|---|---|---|---|
| Ub(11AllocK)$_{His6}$ produced from *Ub(11TAG)$_{His6}$* | N$^{+}$-*Mm* | *Met* | 37.4 ± 0.1 | 76 |
| | A$^{\Delta}$-*1R26* | *Alv*-21 | 36.9 ± 2.9 | 75 |
| | C$^{\Delta}$-*Nitra* | *Therm1* | 31.0 ± 2.1 | 63 |
| | S$^{+}$-*Deb* | *I2*-S52 | 22.7 ± 0.7 | 46 |
| | B$^{\Delta}$-*Lum1* | *I2*-B72 | 43.7 ± 2.3 | 89 |
| Ub(11Ser)$_{His6}$ produced from *Ub(11TCA)$_{His6}$* | N$^{+}$-*Mm* | *Alv*-21 | 49.3 ± 1.6 | 100 |

**Supplementary Table 6**

Peak lists of all mass spectrometry data acquired in this work. The table is provided as a separate excel sheet.

**Supplementary Table 7**

Primary data of protein yield determination from **Supplementary Tables 4** and **5**. The table is provided as a separate excel sheet.

## References


1 Cervettini, D. *et al.* Rapid discovery and evolution of orthogonal aminoacyl-tRNA synthetase–tRNA pairs. *Nat. Biotechnol.*, 1–11 (2020). https://doi.org:10.1038/s41587-020-0479-2

2 Dunkelmann, D. L., Willis, J. C. W., Beattie, A. T. & Chin, J. W. Engineered triply orthogonal pyrrolysyl–tRNA synthetase/tRNA pairs enable the genetic encoding of three distinct non-canonical amino acids. *Nature Chemistry* **12**, 535-544 (2020). https://doi.org:10.1038/s41557-020-0472-x

3 Willis, J. C. W. & Chin, J. W. Mutually orthogonal pyrrolysyl-tRNA synthetase/tRNA pairs. *Nature Chemistry* **10**, 831-837 (2018). https://doi.org:10.1038/s41557-018-0052-5

4 Srinivasan, G., James, C. M. & Krzycki, J. A. Pyrrolysine Encoded by UAG in Archaea: Charging of a UAG-Decoding Specialized tRNA. *Science* **296**, 1459--1462 (2002). https://doi.org:10.1126/science.1069588

5 Krzycki, J. A. The direct genetic encoding of pyrrolysine. *Curr. Opin. Microbiol.* **8**, 706--712 (2005). https://doi.org:10.1016/j.mib.2005.10.009

6 Neumann, H., Peak-Chew, S. Y. & Chin, J. W. Genetically encoding Nε-acetyllysine in recombinant proteins. *Nat. Chem. Biol.* **4**, 232 (2008). https://doi.org:10.1038/nchembio.73

7 Wang, L., Brock, A., Herberich, B. & Schultz, P. G. Expanding the Genetic Code of Escherichia coli. *Science* **292**, 498--500 (2001). https://doi.org:10.1126/science.1060077

8 Borrel, G. *et al.* Unique Characteristics of the Pyrrolysine System in the 7th Order of Methanogens: Implications for the Evolution of a Genetic Code Expansion Cassette. *Archaea* **2014**, 11 (2014). https://doi.org:10.1155/2014/374146

9 Park, H.-S. *et al.* Expanding the Genetic Code of Escherichia coli with Phosphoserine. *Science* **333**, 1151--1154 (2011). https://doi.org:10.1126/science.1207203

10 Rogerson, D. T. *et al.* Efficient genetic encoding of phosphoserine and its nonhydrolyzable analog. *Nat. Chem. Biol.* **11**, 496 (2015). https://doi.org:10.1038/nchembio.1823

11 Hughes, R. A. & Ellington, A. D. Rational design of an orthogonal tryptophanyl nonsense suppressor tRNA. *Nucleic Acids Research* **38**, 6813-6830 (2010). https://doi.org:10.1093/nar/gkq521

12 Chatterjee, A., Sun, S. B., Furman, J. L., Xiao, H. & Schultz, P. G. A Versatile Platform for Single- and Multiple-Unnatural Amino Acid Mutagenesis in Escherichia coli. *American Chemical Society* (2013). https://doi.org:10.1021/bi4000244

13 Fan, C., Xiong, H., Reynolds, N. M. & Söll, D. Rationally evolving $tRNA^{Pyl}$ for efficient incorporation of noncanonical amino acids. *Nucleic Acids Research* **43**, e156-e156 (2015). https://doi.org:10.1093/nar/gkv800

14 Nozawa, K. *et al.* Pyrrolysyl-tRNA synthetase–tRNA$^{Pyl}$ structure reveals the molecular basis of orthogonality. *Nature* **457**, 1163 (2008). https://doi.org:10.1038/nature07611

15 Meineke, B., Heimgärtner, J., Lafranchi, L. & Elsässer, S. J. Methanomethylophilus alvus Mx1201 Provides Basis for Mutual Orthogonal Pyrrolysyl tRNA/Aminoacyl-tRNA Synthetase Pairs in Mammalian Cells. *ACS Chemical Biology* **13**, 3087-3096 (2018). https://doi.org:10.1021/acschembio.8b00571

16 Meineke, B., Heimgärtner, J., Eirich, J., Landreh, M. & Elsässer, S. J. Site-Specific Incorporation of Two ncAAs for Two-Color Bioorthogonal Labeling and Crosslinking of Proteins on Live Mammalian Cells. *Cell Reports* **31**, 107811 (2020). https://doi.org:10.1016/j.celrep.2020.107811

17 Ambrogelly, A. *et al.* Pyrrolysine is not hardwired for cotranslational insertion at UAG codons. *Proc. Natl. Acad. Sci. U.S.A.* **104**, 3141--3146 (2007). https://doi.org:10.1073/pnas.0611634104

18 Zhang, H. *et al.* The tRNA discriminator base defines the mutual orthogonality of two distinct pyrrolysyl-tRNA synthetase/tRNAPyl pairs in the same organism. *Nucleic Acids Research* **50**, 4601-4615 (2022). https://doi.org:10.1093/nar/gkac271

19 Suzuki, T. *et al.* Crystal structures reveal an elusive functional domain of pyrrolysyl-tRNA synthetase. *Nat. Chem. Biol.* **13**, 1261 (2017). https://doi.org:10.1038/nchembio.2497

20 Herring, S. *et al.* The amino-terminal domain of pyrrolysyl-tRNA synthetase is dispensable in vitro but required for in vivo activity. *FEBS Lett.* **581**, 3197--3203 (2007). https://doi.org:10.1016/j.febslet.2007.06.004
